# Hyperuricemia Increases the Risk of Atrial Fibrillation: A Systematic Review and Meta-Analysis

**DOI:** 10.1155/2022/8172639

**Published:** 2022-08-21

**Authors:** Zheng Gao, Hekai Shi, Wei Xu, Zhengzhao Guan, Xiuxiu Su, Nuojin Guo, Huijie Ma

**Affiliations:** ^1^Department of Physiology, Hebei Medical University, Shijiazhuang, China; ^2^Department of First Affiliated Hospital of Hebei Medical University, Shijiazhuang, China; ^3^Department of Second Affiliated Hospital of Hebei Medical University, Shijiazhuang, China; ^4^Rizhao Campus, Qufu Normal University, Qufu, China; ^5^College of Physical Education, Hebei Normal University, Shijiazhuang, China; ^6^Department of Third Affiliated Hospital of Hebei Medical University, Shijiazhuang, China

## Abstract

Association between hyperuricemia (HUA) and atrial fibrillation (AF) remains unclear. We reviewed clinical evidence and aimed to determine whether hyperuricemia leads to a high risk of atrial fibrillation. Most studies were identified through databases online. Keywords used in literature search were hyperuricemia, atrial fibrillation, metabolic disorder, endocrine disorder, or uric acid. Three studies were provided by the authors. Literature search was performed without any data or language restriction. Observational studies, including cohort studies and cross-sectional studies, were used. Study type should be clearly defined. Cross-sectional studies should clearly introduce the sources of epidemiological data. Studies were excluded if with too many complications unrelated to AF enrolled. Data were independently extracted by three individuals. Data synthesis was conducted by R version 4.1.2. Prevalence of atrial fibrillation was the main outcome. Results of meta-analysis were presented as risk ratio (RR) for different prevalence of AF between individuals with and without HUA. All data included were obtained after follow-up work is completed. Data from 608,810 participants showed that patients with hyperuricemia were easier to suffer from atrial fibrillation (RR, 2.42; 95% CI, 1.24–3.03). And the meta-regressions suggested growth of linear proportion between the ratio of current drinkers and hyperuricemia (QM = 41.0069, *P* < 0.001). Subgroup analyses demonstrated consistent results in different countries. And design of the observational studies brought heterogeneity, but no uncertainties. Patients with hyperuricemia were easier to suffer from atrial fibrillation. Treatment of hyperuricemia or gout may bring potential benefits for AF patients.

## 1. Introduction

Hyperuricacidemia (HUA) is a metabolic disease caused by purine metabolism and imbalance of uric acid production and excretion [1], which was defined as a uric acid level >7.0 mg/dL in men and >5.7 mg/dL in women [2]. HUA was also reported as the mean cause of gout, which helps to trigger atrial fibrillation (AF) and lead to other cardiac diseases [3]. It is reported that the prevalence of AF is increasing worldwide [4], and treatment, prevention, and detection of atrial fibrillation is becoming a hotspot of epidemiology, neurology, and cardiovascular diseases [5]. Recent studies have reviewed the difficult relationship between uric acid (UA) and cardiovascular diseases [6–8]. And it was confirmed that serum uric acid (SUA) levels are related to atrial fibrillation [9], a critical risk factor of cerebrovascular [10] and cardiovascular events [11, 12]. Studies have reported a higher risk in persistent AF compared with paroxysmal AF [13–15]. Falk predicted that pharmacologic therapy still acts as the mainstay of treatment of AF [16], which means not only antiarrhythmic drugs, anticoagulants, or vitamin K antagonists. Gupta and Singh [17] reviewed the clinical evidence of the association between SUA and AF, and suggested treatment of hyperuricemia may benefit on AF patients, respectively. However, the association between HUA and AF remains unclear, for which the present meta-analysis systematically reviewed clinical evidence and may give suggestions for therapy of those AF patients with gout or HUA.

## 2. Results

**Figure 1:**
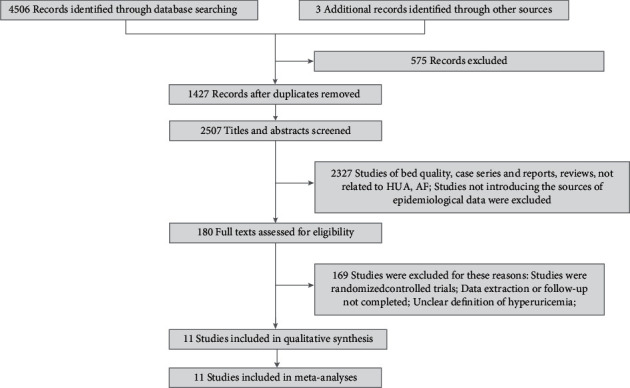
PRISMA flow diagram summarizing the article selection process.

Among the 4509 studies identified, 11 observational studies [18–28] with a total of 608,810 participants were included for the present meta-analysis. Literature search and identification were summarized in a flow chart (Figure 1). A total of 5 (45.45%) studies were cross-sectional (involving 114,819 individuals) [18, 19, 21, 22, 24], 4 (27.27%) were representative cohort studies (involving 486,559 individuals) [20, 23, 25, 28], and 2 (18.18%) were prospective cohort studies (involving 7432 individuals) [26, 27]. Most of the studies provided a date range of case selection. Six (54.55%) studies took place in China [18–20, 22, 23, 25], 2 (18.18%) studies in Japan [21, 28], whereas 1 (9.09%) study occurred in the United States [27], Italy [26], and Poland [24], respectively. Following characteristics of studies were extracted: age, sex proportion, BMI, uric acid level, current drinking proportion, current smoking proportion, location of the study, study design, etc. Medicines use that might have influenced the results of this review was also recorded. Details were described in Table 1.

Of the 11 studies, 1 (9.09%) study was at serious risk of bias, 4 (36.36%) studies were at moderate risk of bias, and 6 (54.55%) studies were at low risk of bias. Studies with two domains of “moderate risk” or one domain of higher risk were thought to be at “moderate risk.” Studies with three or more risks and one or more “serious risks” were thought to be at “serious risk.” Studies with two or more “critical risks” were excluded. And a comprehensive assessment of the included studies suggests the present evidence of medium credibility. Details were shown in Supplement Figures 1 and 2.

**Figure 2:**
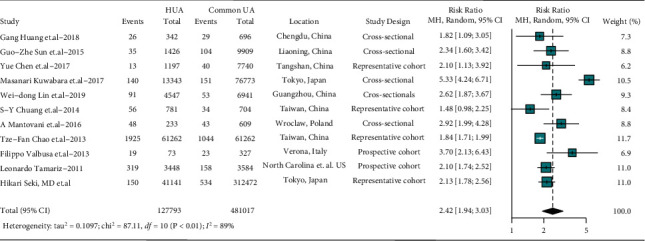
Difference in prevalence of AF between those with or without HUA. Point sizes are an inverse function of the precision of the estimates, and bars correspond to 95% CIs; HUA: hyperuricemia, common UA: individuals without HUA.

Pooled data showed that patients with hyperuricemia were easier to suffer from atrial fibrillation (RR, 2.42; 95% CI, 1.24–3.03), with a significant heterogeneity (*I*^2^ = 89%, *P* < 0.01), as shown in the forest plot (Figure 2). And the meta-regressions suggested growth of linear proportion between the ratio of current drinkers and hyperuricemia (*P* < 0.001), considering as a possible source of heterogeneity (Supplement Figure 3). Age, male proportion, uric acid level, smoking, and body mass index (BMI) make no significant influence. Regression curve of covariates were described in Supplement Figure 4(a)–4(f).

**Figure 3:**
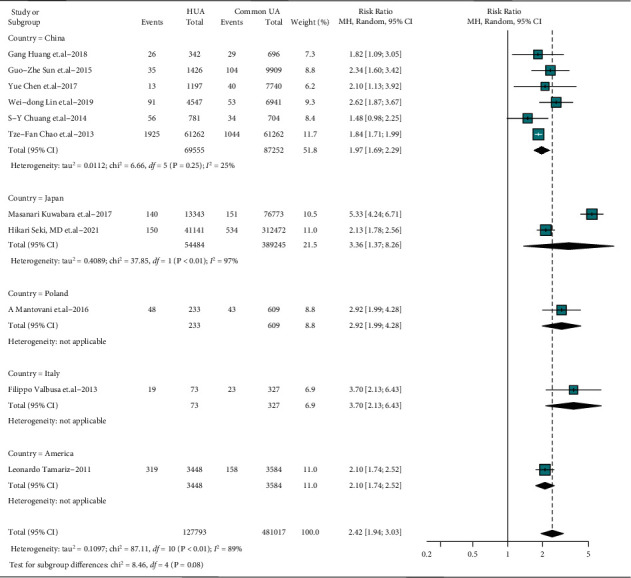
Associations between AF and HUA in different countries. Point sizes are an inverse function of the precision of the estimates, and bars correspond to 95% CIs; HUA: hyperuricemia, common UA: individuals without HUA.

**Figure 4:**
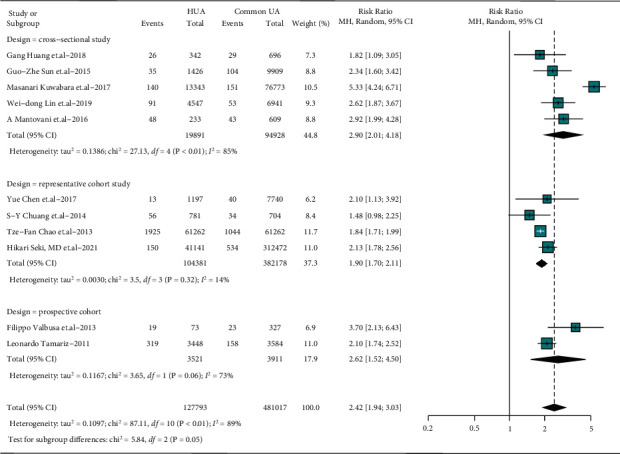
Associations between AF and HUA in studies of different designing. HUA: hyperuricemia, common UA: individuals without HUA; significance was set as *P* < 0.05.

Studies of different designs may provide evidence of different intensities, and studies enrolling individuals of various races bring heterogeneity and uncertainties. Studies from different countries and designs were subgrouped and meta-analyzed, results of which were all consistent with the main comprehensive meta-analysis (country subgroup: RR = 1.97–3.70, detective heterogeneity *I*^2^ = 25–97%, *P* < 0.05; study design subgroup: RR = 1.83–2.90, detective heterogeneity *I*^2^ = 0–85%, *P* < 0.05), as shown in Figures 3 and 4. The subgroup analyses suggest countries and designs sources of heterogeneity, expected as described previously.

As shown in Supplement Figure 5 and Table 1, sensitivity analysis suggests a stable and credible conclusion of the present meta-analysis. Significant publication bias was not observed (Peter's test; *t* = 1.45, *P*=0.1824) (Supplement Figures 6 and 7). And to investigate whether the use of renin-angiotensin-aldosterone system (RAAS) blockers will affect this review's result and explore the role of the RAAS, we performed a subgroup analysis of studies available on the use of RAAS blockers. Subgroup analysis was conducted based on the proportion of RAAS blockers users greater than or less than 50%. The result suggested that RAAS blockers have no significant effect on the results of the present review (RR, 2.70; 95% CI, 1.87–3.90), same as described in Supplement Figure 8. As shown in Table 1, eGFR values suggest a general renal insufficiency of subjects in each study. Hence a meta-regression was performed based on eGFR values. The result indicated that differences in renal function were not one of the primary sources of heterogeneity and did not significantly affect the results of the present meta-analysis (Supplement Figure 9).

The number of participants taking angiotensin-converting enzyme inhibitor and angiotensin receptor blockers (ACEI/ARB), calcium channel blockers (CCB), *β* receptor blockers, and diuretics were recorded and provided in Table 1. And as shown by the sensitivity analysis and Table 1, SUA-affecting drugs used at different doses did not significantly affect the results of this review.

## 3. Discussion

The present meta-analysis synthesized data from 11 observational studies with a total of 608,810 participants enrolled, and drew a conclusion that patients with HUA were more likely to suffer from AF than those without HUA. And the chief strength of the present meta-analysis was the huge simple size and consistency of participants. To our knowledge, this is the first meta-analysis to confirm the association between HUA and AF, as well as the largest systematic review ever published. And compared with those previous relative systematic reviews, the types of studies we included were more consistent. These make the evidence we provided more credible. In previous studies, some have reported the association between uric acid level and AF, which have suggested a potential close relation [29, 30]. We further proved the impact HUA has on AF when SUA level was higher than 7.0 mg/dL in men and 5.7 mg/dL in women [2]. And this has important treatment ramifications for the general and cardiologist, providing them with credible evidence for using medicines such as allopurinol [31] or benzbromarone [32] for those AF patients with HUA or gout.

The chief mechanism of hyperuricemia has been thought to be the accumulation of uric acid caused by purine metabolic disorder. However, insufficient excretion of uric acid was considered to play a central role in the pathogenesis of hyperuricemia more recently due to the finding of the key role of proximal tubules in uric acid absorption. Gout syndrome is the main clinical manifestation and belongs to a hereditary disease [33, 34]. A recent Italian study [35] reported that the overall incidence of AF in the elderly aged ≥65 years old without known AF or anticoagulant therapy was 5.5%, of which 3.6% was in the 65–74 years old and 7.5% was in the ≥75 years old. This suggests elder individuals are easier to suffer from AF. However, the present review shows that a high prevalence rate of HUA may lead to a younger distribution of AF, which suggests the essentiality of HUA treatment. As Falk said in the *New England Journal of Medicine*, medicines appear to remain the chief treatment for AF in the short term. And a comprehensive treatment for AF was also recommended [16]. We suggest that treatment of complications may have potential benefits for atrial fibrillation. Frequently or commonly prescribed drugs may lower or raise SUA levels, including atorvastatin, calcium channel blockers, alpha-1-adrenergic antagonists, sevelamer, metformin, angiotensin-II/neprilysin inhibitors, fenofibrate, theophylline, and so on [36–44]. In particular, medicines such as statins that cause elevated SUA levels need to be used more carefully, especially in patients at high risk of cardiovascular disease.

In the present meta-analysis, HUA was significantly correlated with the incidence of AF. Moreover, significant heterogeneity was detected between the results of patients with atrial fibrillation and those without atrial fibrillation. Meta-regressions (including average age, BMI, the proportion of male patients, proportion of current smoking) failed to explain this heterogeneity. The proportion of current drinking was considered as a potential source of heterogeneity and a potential covariate, which was consistent with other studies [45, 46]. And the heterogeneity is considered predictable due to the diversity of patients, including the variability of atrial fibrillation types. However, many studies including systematic reviews have reported the close association of smoking with AF [47–49]. It is worth noting that a large national nutritional health survey in South Korea has revealed a close relationship between smoking and SUA in female but not in male subjects [50]. The present meta-analysis failed to determine the influence of smoking on the HUA's promotion of AF for the lack of sample size and statistical appearance, which may need more evidence. And the eGFR values suggest a general renal insufficiency of subjects in most studies. But the result of the meta-regression indicated that differences in renal function were not one of the primary sources of heterogeneity and did not significantly affect the results of the present meta-analysis.

Uric acid level has been reported closely related to cardiac diseases, which enhances oxidative stress, disrupts cardiovascular function, promotes inflammations, increases insulin resistance, and activates the RAAS [51–56]. Although not entirely clear, the mechanism pathogenesis of AF can be summarized as atrial fibrosis, inflammations, oxidative stress, and dysfunction of RAAS [57–60], almost parallel to mechanisms mentioned previously. The subgroup analysis suggested that the use of RAAS blockers had no significant effect on the result of this review. This indicated that RAAS might have less weight in increased incidence of AF caused by HUA.

It was noted that Deng et al. [61] had drawn a similar conclusion with the present meta-analysis in an article review recently, which suggested hyperuricemia as an important risk factor contributing to atrium injury and AF. Compared to Deng et al.'s review, the present review provides more credible evidence which supports his point.

The present meta-analysis has some limitations. The large amount of unexpected heterogeneity is the most notable. Results of sensitivity analysis and meta-regression failed to detect the possible source of heterogeneity. This is likely attributable to variability in study design (including source of data, duration of follow-up), sociodemographic factors, measure tools, frequency and type of testing, local policies, or natural environment. Ethnic differences are considered one of the main sources of heterogeneity, for the various heterogeneity and outcomes in different subgroups. Drugs affecting subjects' SUA levels may also have introduced some heterogeneity. Moreover, due to the lack of information on drug use, meta-regression could not be performed to explore the effects of drugs affecting subjects' SUA levels. Unrelated complications were not rolled out in most studies except for Chen et al. [20] and Kuwabara et al. [21], which bring both heterogeneity and uncertainty.

Important questions remain regarding AF and HUA. Chief among them is lack of cohort studies or random controlled trials reporting changes in the prevalence of atrial fibrillation after treatment of hyperuricemia. Data were not available to confirm the effect of uric acid lowing on the prevalence of AF, which requires more studies.

The findings of this study suggest that hyperuricemia is a risk factor of AF, and treatment for hyperuricemia can bring potential benefits for AF patients.

## 4. Methods

### 4.1. Search Strategy

Following guidance of Cochrane handbook [62] for systematic review and the Preferred Reporting Items for Systematic Reviews and Meta-analyses (PRISMA) [63], we searched in PubMed, Clinical trial, Embase database, Cochrane library, China national knowledge infrastructure (CNKI), Epigraph DB (https://epigraphdb.org/), DigiZeitschriften (https://www.digizeitschriften.de/), and Web of Science with hyperuricemia, atrial fibrillation, metabolic disorder, endocrine disorder, or uric acid (Supplement Appendix 1). Literature search was performed on November 17, 2021, without any data or language restriction. And the present review has been registered in the International Platform of Registered Systematic Review and Meta-analysis Protocols (INPLASY) (INPLASY2021120092; DOI: 10.37766/inplasy2021.12.0092).

### 4.2. Eligibility Criteria

We excluded studies that may lead to an incredible conclusion. We included cross-sectional, retrospective, and prospective cohort studies. Randomized controlled trials will be excluded. The reason is that randomized controlled trials are too rare to lead to independently certain results, as introduced later. And difference in study designing will bring uncertainty to the evidence provided in this study.

Study type should be clearly defined. Cross-sectional studies should clearly introduce the sources of epidemiological data. Sources as public health databases, hospital medical records, or insurance agencies were acceptable. The number of participants should be sufficient to avoid contingency. Any study of participants less than 200 will be excluded. Studies were excluded if with too many complications unrelated to AF enrolled. And patients with significantly reduced renal function were not used in this review. We included only studies that all the follow-up work completed to ensure the whole data were available. Criteria of hyperuricemia or AF should be defined clearly, which should be consistent with the universal knowledge. Studies that got “critical risk” in overall were excluded (Supplement Figure 1 PRISMA flow chart). Retrospective studies reporting the association between AF and HUA were included. Participants should be defined with hyperuricemia directly. Case reports, protocols, or trials with incomplete follow-up were excluded.

### 4.3. Data Extraction

Data of each trial were extracted directly from the literature. Literature identifying and extraction were performed by three reviewers (G.Z., S.H.K., S.X.X.) independently. Any disagreement was reported to another reviewer (X.W.). Details were shown in Supplement 1, search details.

### 4.4. Evaluation of Study Quality and Risk of Bias

Quality evaluation was operated by R version 4.1.2 using the Risk Of Bias In Non-randomised Studies-of Interventions (ROBINS-I) [64]. One reviewer (G.Z.) evaluated all studies included in seven domains: (1) Bias due to confounding; (2) bias due to selection of participants; (3) bias in classification of interventions; (4) bias due to deviations from intended interventions; (5) bias due to missing data; (6) bias in measurement of outcomes; (7) bias in selection of the reported results, and labeled them as “low risk,” “moderate risk,” “serious risk,” and “critical risk.” Details of quality evaluation appear in Supplement Figures 1 and 2.

### 4.5. Statistical Analysis

Following the retrieval format of PICO [65], two of the reviewers (G.Z, S.H.K.) extracted the characteristics of each study included. Details are described in Table 1 (Characteristics of studies). Statistical analyses were performed by one reviewer (G.Z.) and double checked by one reviewer (S.H.K.). We compared the proportion of atrial fibrillation in people with and without hyperuricemia. AF was detected in physical examination or follow-up. An epidemiologist (X.W.) evaluated quality of the cross-sectional studies and rectified the data based on Cochrane' s handbook, for a better applicability of the present review.

Meta-analysis was operated on R version 4.1.2 using the package “meta” (R Project for Statistical Computing) (R Core Team. R: a language and environment for statistical computing. Vienna R Foundation for Statistical Computing; 2019. https://www.R-project.org). Risk ratio (RR) and its 95% confidence interval were used to evaluate the proportion of atrial fibrillation in people with and without hyperuricemia. Studies were subgrouped and regressed to detect possible sources of heterogeneity and potential influencing factors of AF proportion. All statistical effects were calculated in a random effect model, with a two-tailed *α* = 0.05 set as the statistical significance. The Cochrane's *Q* test and *I*^2^ were calculated as measures of heterogeneity. *I*^2^ values of 25%, 50%, and 75% were thought to indicate a low, moderate, or high heterogeneity [66], which we took into account when conducting the data synthesis. Details are described in Supplement 3, Statistical Analysis. A professional engineer (G.Z.Z.) helped adjusting the R project's packages to make the Fisher algorithms more applicable to this review.

Sensitivity analysis was performed to confirm the reliability and detect potential sources of heterogeneity. Deek's funnel plots and Peter's test were used to detect publication bias. Potential publication bias would be corrected using the method “trim and fill” [67], and publication bias was thought to exist if corrected results challenge previous conclusions.

## Figures and Tables

**Table 1:** Characteristics of the included studies.

Author	Year	Participants	Location	Average age	Average SUA	Sex proportion (male)	Current drinking	ACEI/ARB	CCB	*β*-receptor blocker	Diuretics

Huang et al. [18]	2018	1038	Chengdu, China	83.6 ± 3.4	350.1 ± 84.5 *μ*mol/L	N	8.30%	11.90%	26.1%	7.8%	6.6%
Sun et al. [19]	2015	11,335	Liaoning, China	58.22 ± 11.74	367.20 ± 98.39 *μ*mol/L	35.20%	N	N	N	N	N
Chen et al. [20]	2017	8937	Tangshan, China	42.1 ± 13.1	5.0 ± 1.5 mg/dL	52.40%	33.20%	N	N	N	0.6
Kuwabara et al. [21]	2017	90,116	Tokyo, Japan	46.35 ± 13.1	5.28 ± 1.5 mg/dL	49.12%	62.03%	N	N	N	N
Lin et al. [22]	2019	11,488	Guangzhou, China	58.22 ± 11.74	367.20 ± 98.39 *μ*mol/L	35.20%	21.50%	N	N	N	N
Chuang et al. [23]	2014	1485	Taiwan, China	71.87 ± 11.74	6.63 mg/dL	51.18%	N	N	N	N	2.76%
Mantovani et al. [24]	2016	842	Wroclaw, Poland	66.08 ± 13.1	5.44 ± 1.5 mg/dL	55.14%	N	53.77%/20.55%^*∗*^	32.56%	31.26%	50.47%
Chao et al. [25]	2013	122,524	Taiwan, China	49.06 ± 11.74	5.97 ± 1.5 mg/dL	62.89%	N	N	N	N	N
Valbusa et al. [26]	2013	400	Verona, Italy	63.63 ± 11.74	307.88 ± 98.39 *μ*mol/L	58.71%	N	31%	14.5%	6.25%	14%
Tamariz et al. [27]	2011	7032	North Carolina, Mississippi, Mississippi, Mississippi, USA	N	N	N	N	N	N	N	17.53%
Seki et al. [28]	2021	353,613	Tokyo, Japan	39.68 ± 3.4	N	46.87%	17.64%	N	N	N	N

Author		Urate lowering medicines in HUA group (n)			Average eGFR (ml/min/1.73 m^2^)		BMI (kg/m^2^)	Gout	Current smoking	Study design	

Huang et al. [18]	2018	N			58.7		23.1 ± 3.7	N		Cross-sectional	
Sun et al. [19]	2015	N			N		24.01 ± 3.55	N	N	Cross-sectional	
Chen et al. [20]	2017	Statins (23)			97.6		24.5 ± 3.7	0.39%	26.00%	Representative cohort	
Kuwabara et al. [21]	2017	N			85.69		22.41 ± 3.7	N	40.67%	Cross-sectional	
Lin et al. [22]	2019	N			N		24.01 ± 3.55	N	21.30%	Cross-sectional	
Chuang et al. [23]	2014	104 in total			73.66		23.8 ± 3.55	N	22.52%	Representative cohort	
Mantovani et al. [24]	2016	243 in total			64.92		30.32 ± 3.7	N	52.11%	Cross-sectional	
Chao et al. [25]	2013	61,262 in total			82.89		N	N	N	Representative cohort	
Valbusa et al. [26]	2013	ACE inhibitors or sartans (64); calcium channel blockers (32); *β* blockers (15); *α* blockers (8); insulin therapy (21); allopurinol therapy (9).			83.71		29.11 ± 3.55	N	20.58%	Prospective cohort	
Tamariz et al. [27]	2011	N			N		N	N	20.16%	Prospective cohort	
Seki et al. [28]	2021	0			N		21.73 ± 3.7	N	24.12%	Representative cohort	

^N^ None; SUA, serum uric acid; AF, atrial fibrillation; BMI, body mass index; ACEI, angiotensin-converting enzyme inhibitors; ARB, angiotensin receptor blocker; CCB, calcium channel blocker; eGFR, estimate glomerular filtration rate; ^*∗*^ 53.77% used ACEI and 20.55% used ARB. Age, uric acid level, and BMI were described in mean ± standard deviation (SD); current drinking, smoking individuals, gout patients, and diuretic using patients were described in percentages. BMI was described in kg/m^2^.

## Data Availability

No data were used to support this study.

## Additional Points

The title could be found in the registry for protocol as per the following link: https://dspace.bracu.ac.bd:8080/xmlui/bitstream/handle/10361/15963/17336004_MNS.pdf?isAllowed=y&sequence=1.

## Supplementary Materials

Supplementary Materials 1. Supplements. To make our work more organized, some pictures or tables were put in a supplementary material named *Supplements*. All the pictures and tables in the *Supplements* were cited and illustrated in the article. 2. Graphical Abstract Image. A graphical abstract, concise and comprehensive of the main contribution of our work. 3. Graphical Abstract Text. A short explanation of our graphical abstract.
