# Supplementary material for: Hyperuricemia Increases the Risk of Atrial Fibrillation: A Systematic Review and Meta-Analysis
Source: Int J Endocrinol. 2022 Aug 21;2022:8172639. doi: 10.1155/2022/8172639 (PMC9420608; doi:10.1155/2022/8172639)
Supplement: Supplementary Materials — 1. Supplements. To make our work more organized, some pictures or tables were put in a supplementary material named Supplements. All the pictures and tables in the Supplements were cited and illustrated in the article. 2. Graphical Abstract Image. A graphical abstract, concise and comprehensive of the main contribution of our work. 3. Graphical Abstract Text. A short explanation of our graphical abstract. [file 8172639.f1.zip › Graphical_Abstract_Text.pdf]

In this work, we synthesized the epidemiological evidence to determine the associations between hyperuricemia (HUA) and atrial fibrillation (AF). Data was collected from 11 large-scale cross-sectional and cohort studies with a total of 608810 participants enrolled. And we confirmed that patients with hyperuricemia were easier to suffer from AF.
