# Supplementary material for: Hyperuricemia Increases the Risk of Atrial Fibrillation: A Systematic Review and Meta-Analysis
Source: Int J Endocrinol. 2022 Aug 21;2022:8172639. doi: 10.1155/2022/8172639 (PMC9420608; doi:10.1155/2022/8172639)
Supplement: Supplementary Materials — 1. Supplements. To make our work more organized, some pictures or tables were put in a supplementary material named Supplements. All the pictures and tables in the Supplements were cited and illustrated in the article. 2. Graphical Abstract Image. A graphical abstract, concise and comprehensive of the main contribution of our work. 3. Graphical Abstract Text. A short explanation of our graphical abstract. [file 8172639.f1.zip › Supplements (1).pdf]

Supplements

s Figure 1. Summary of risk biases in the included studies.

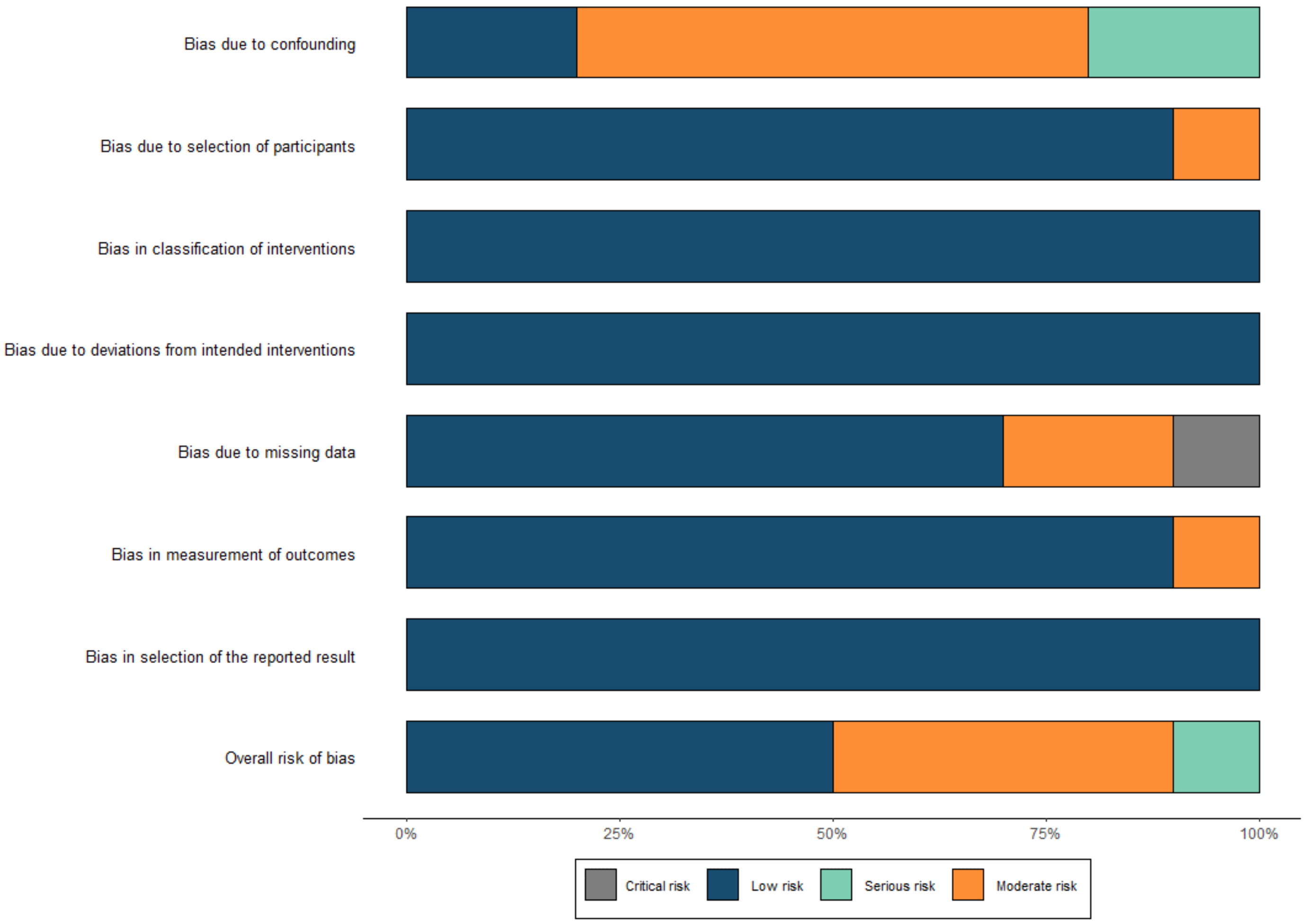

s Figure 2. Risk biases of each study.

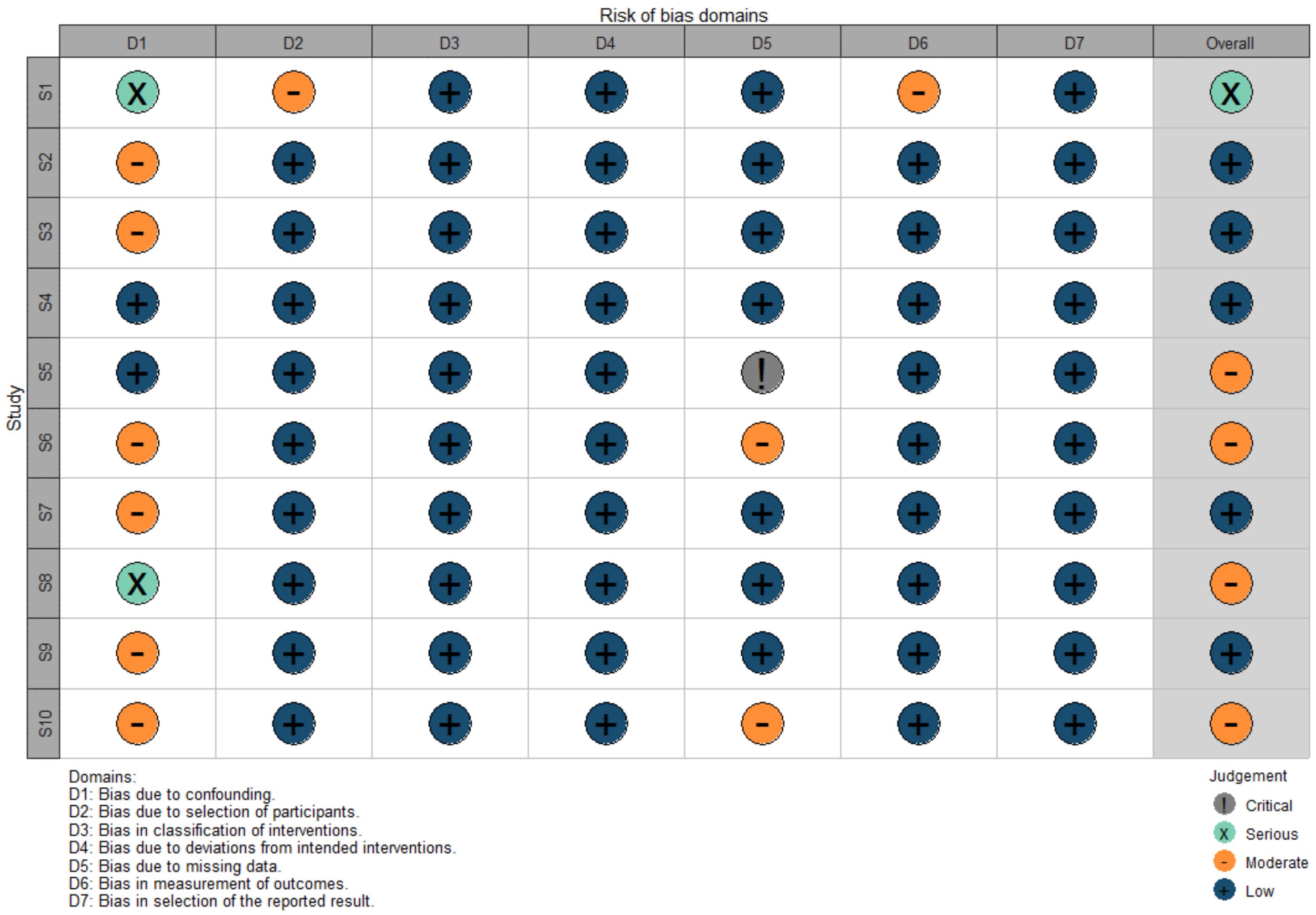

s Fig 3 Meta-regression details of the current drinker's ratio.

```
Mixed-Effects Model (k = 5; tau^2 estimator: REML)

tau^2 (estimated amount of residual heterogeneity):     0 (SE = 0.0232)
tau (square root of estimated tau^2 value):             0
I^2 (residual heterogeneity / unaccounted variability): 0.00%
H^2 (unaccounted variability / sampling variability):   1.00
R^2 (amount of heterogeneity accounted for):            100.00%

Test for Residual Heterogeneity:
QE(df = 3) = 1.6346, p-val = 0.6516

Test of Moderators (coefficient 2):
QM(df = 1) = 41.0069, p-val < .0001

Model Results:

          estimate      se    zval    pval   ci.lb   ci.ub
intrcpt     0.4180  0.1167  3.5831  0.0003  0.1893  0.6466  ***
Drinking    2.0054  0.3132  6.4037  <.0001  1.3916  2.6192  ***

---
Signif. codes:  0 ‘***’ 0.001 ‘**’ 0.01 ‘*’ 0.05 ‘.’ 0.1 ‘ ’ 1
```

s Fig 4A~4F. Regression curves of covariates

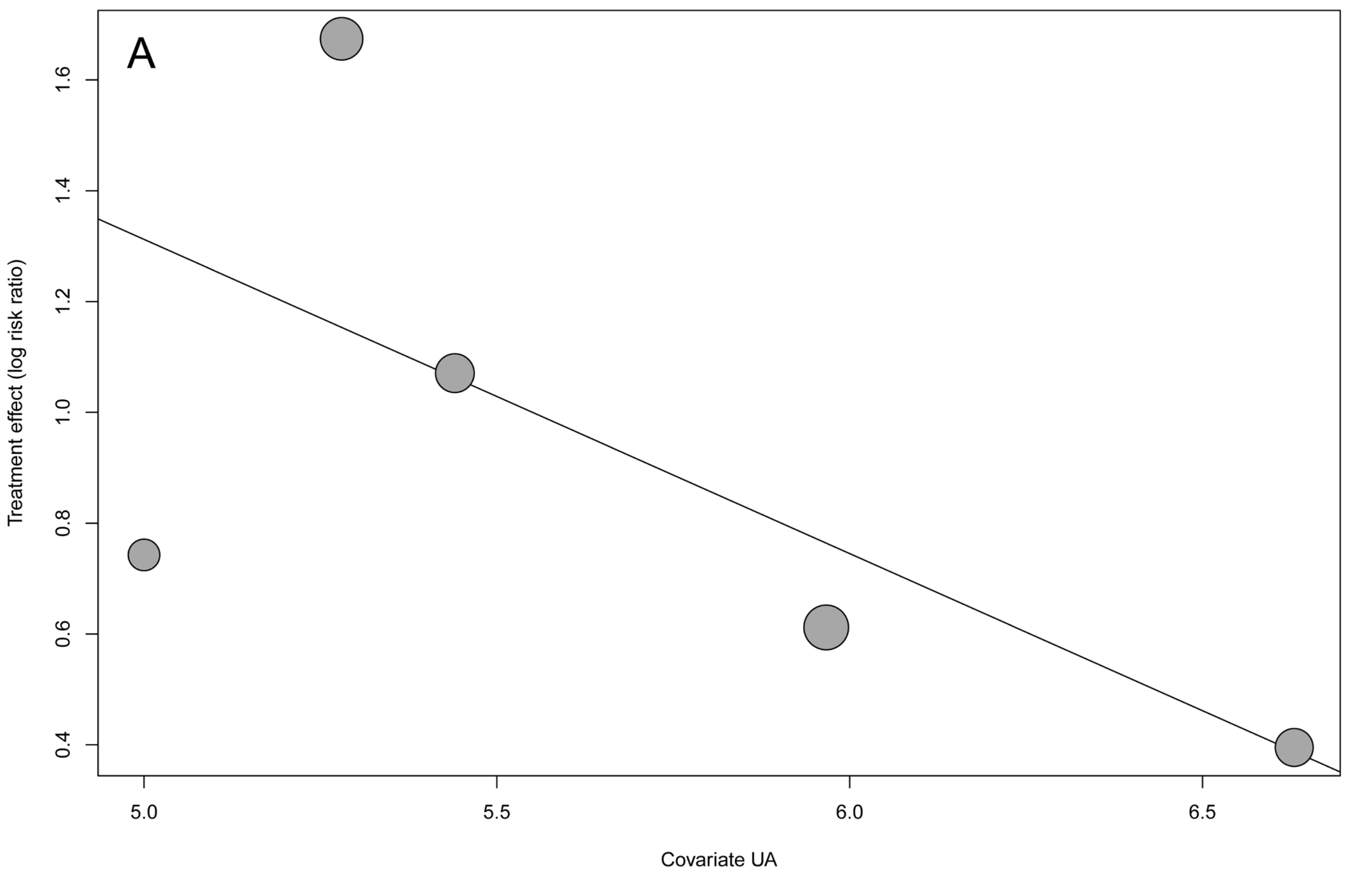

s Fig 4A

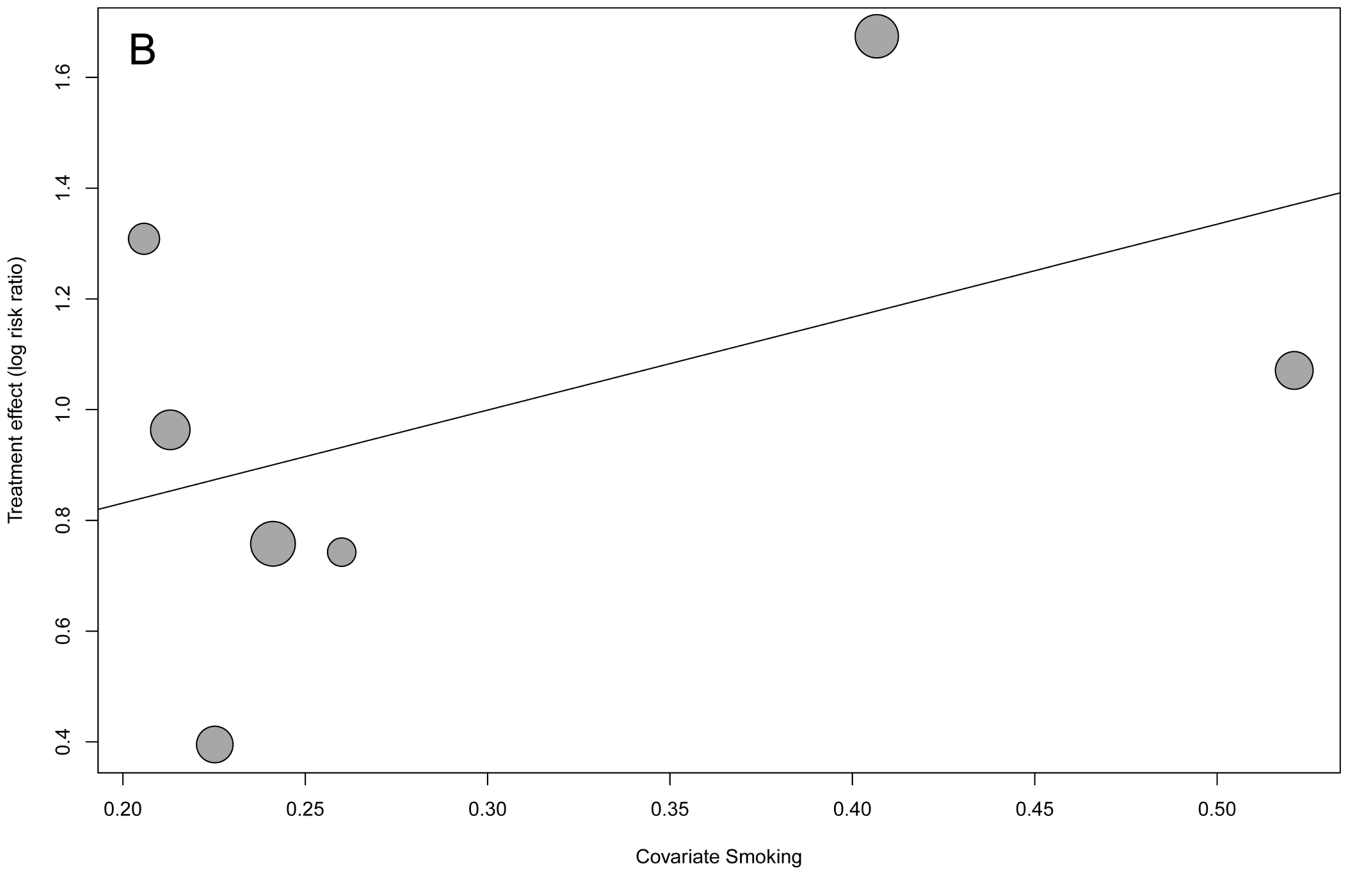

s Fig 4B

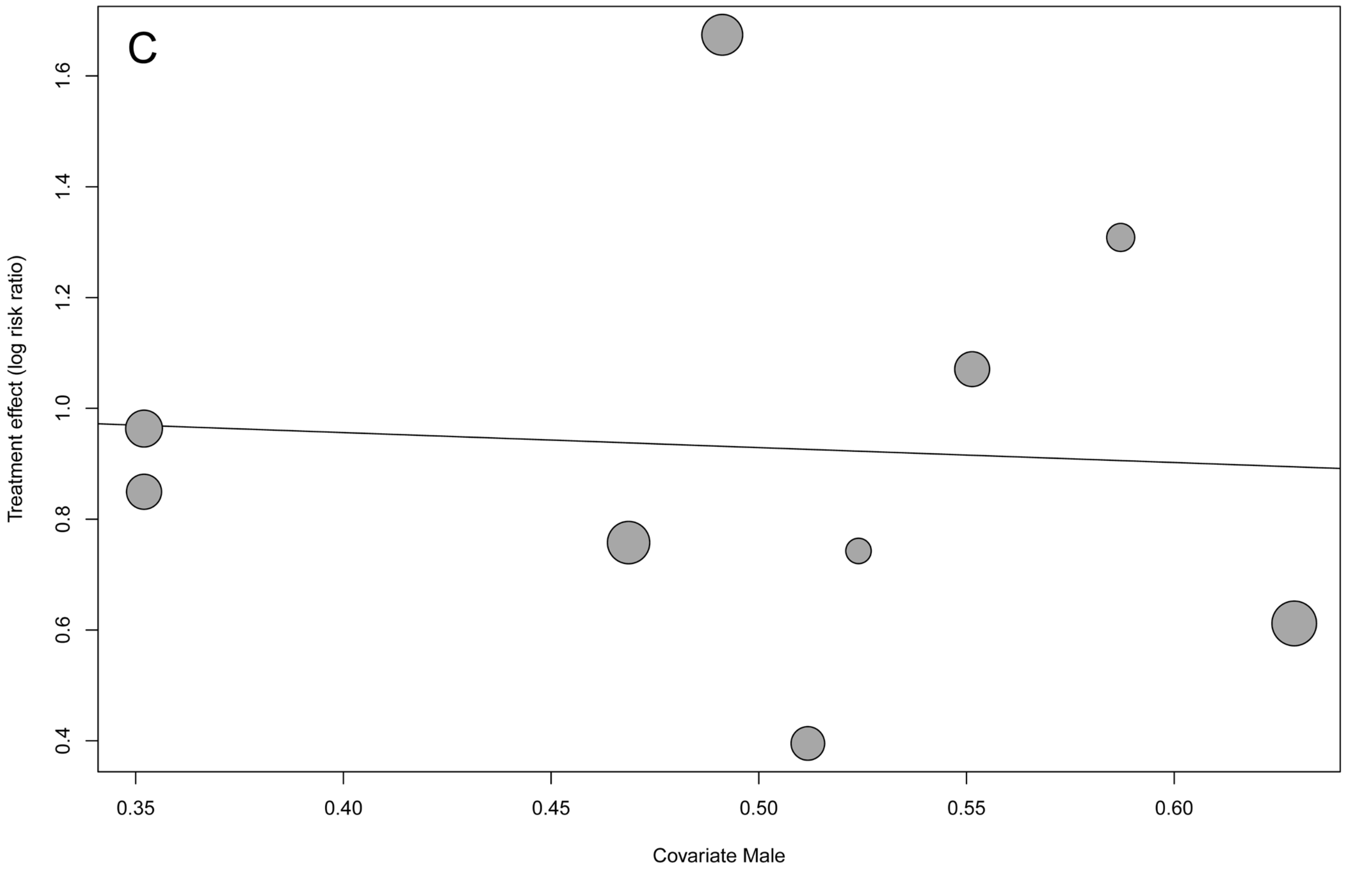

s Fig 4C

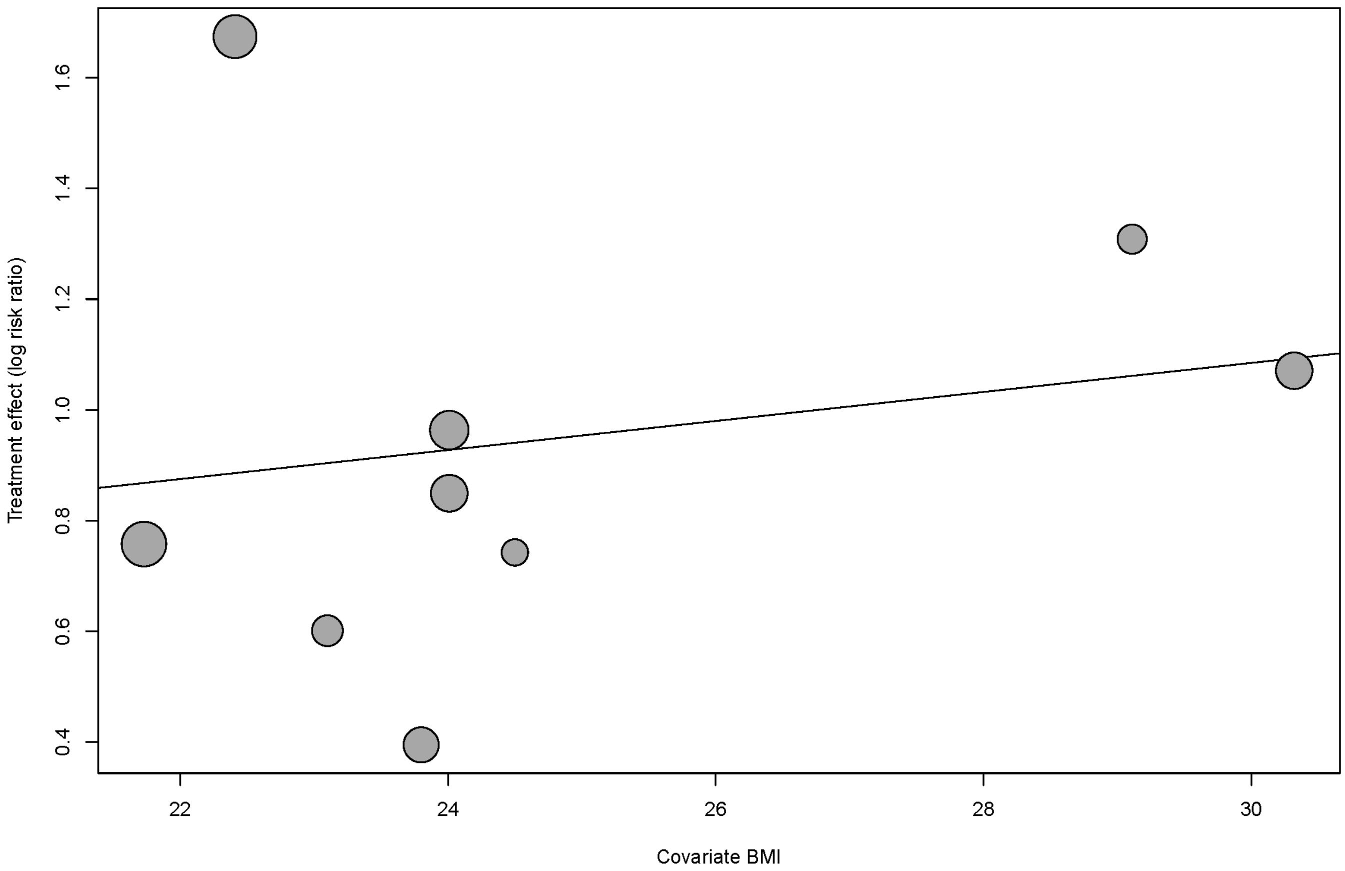

s Fig 4D

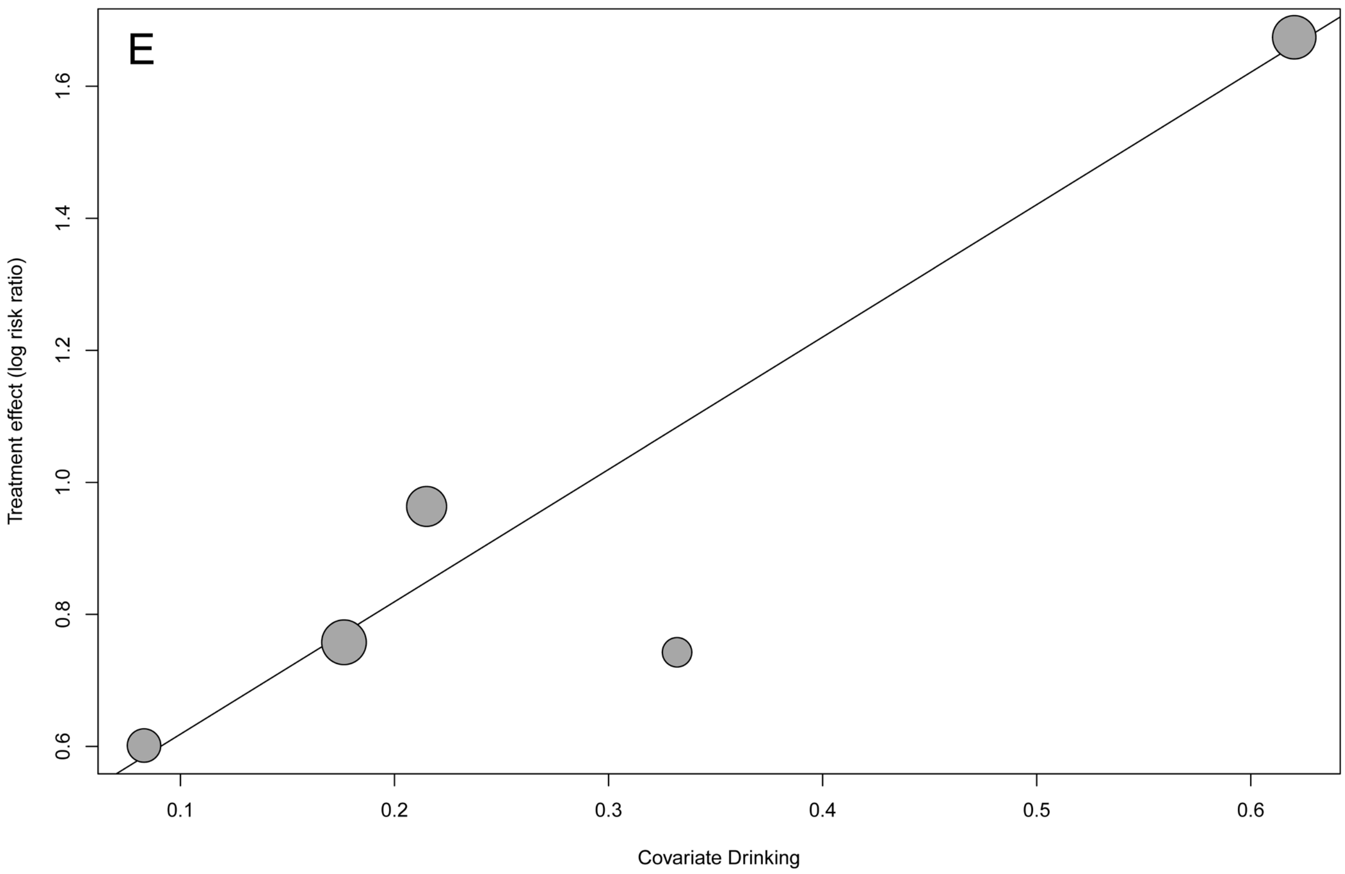

s Fig 4E

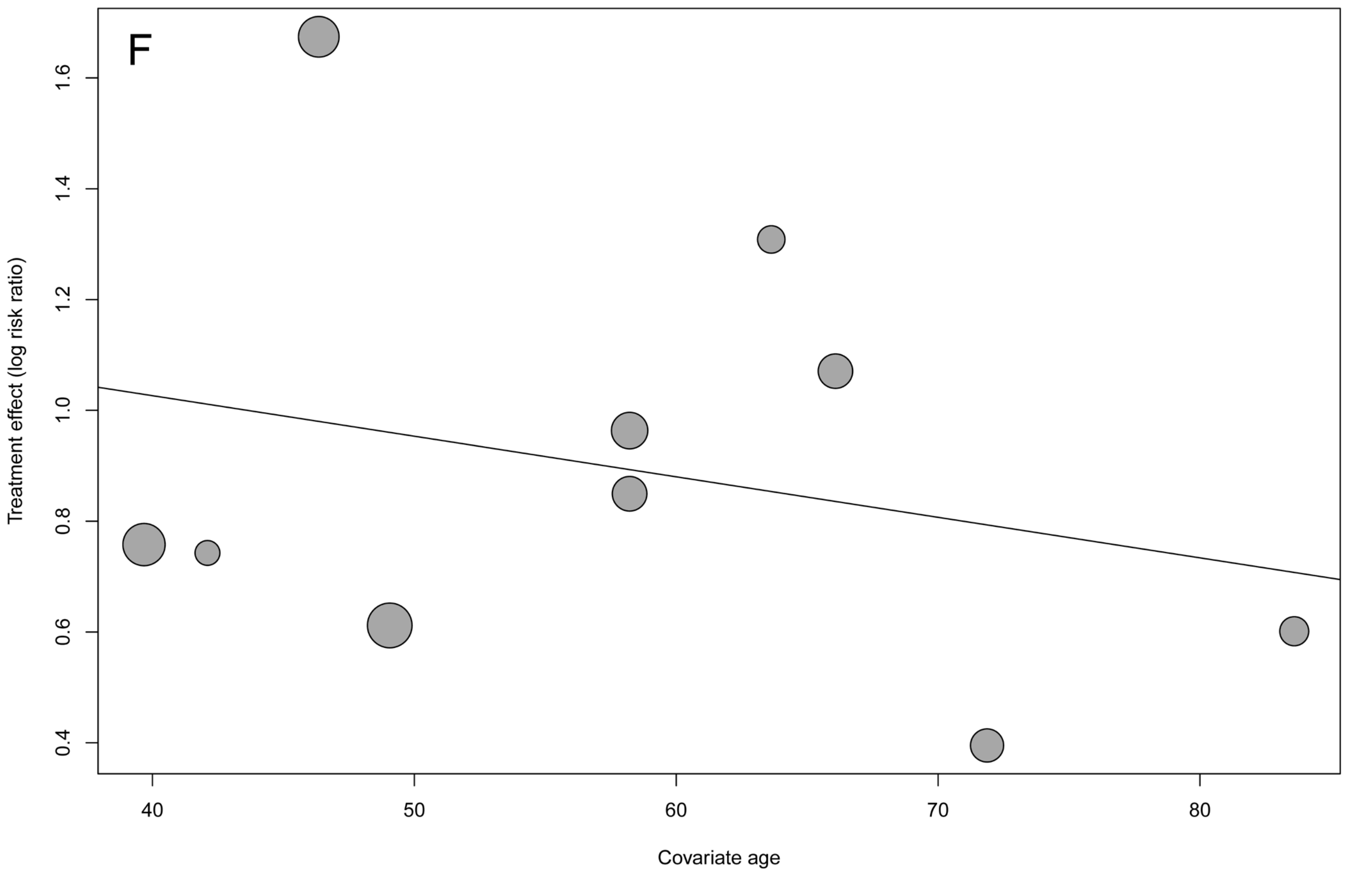

s Fig 4F

s Fig 5. Sensitivity analysis.

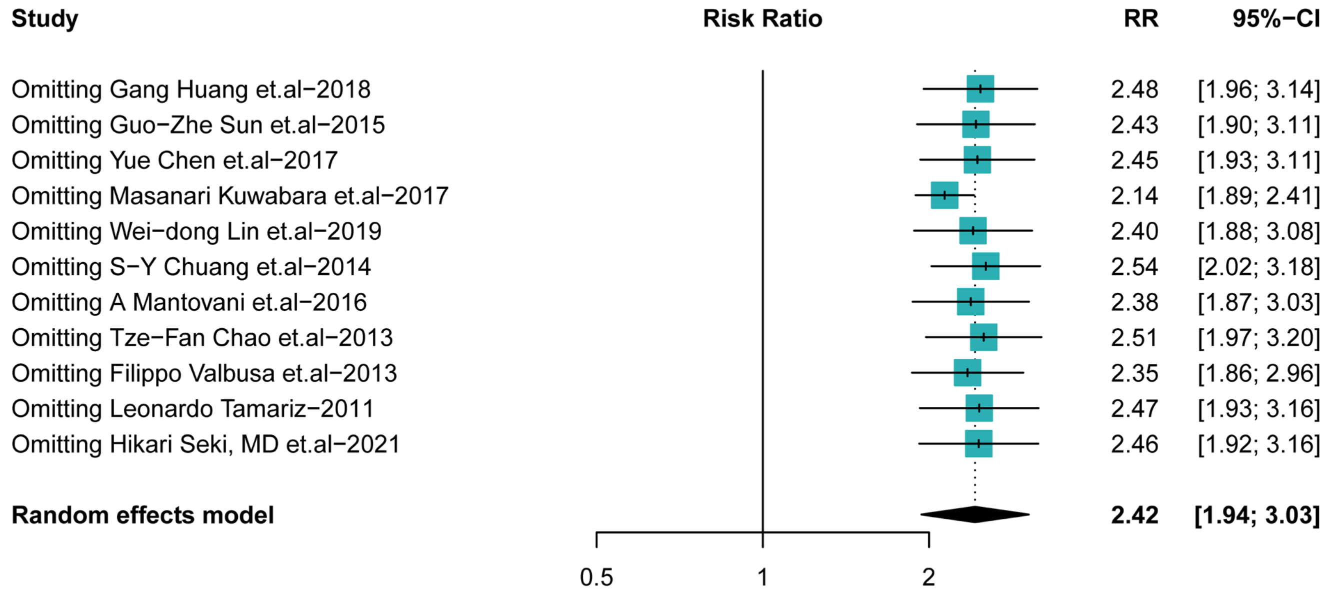

s Fig 6. Deek’s funnel plot

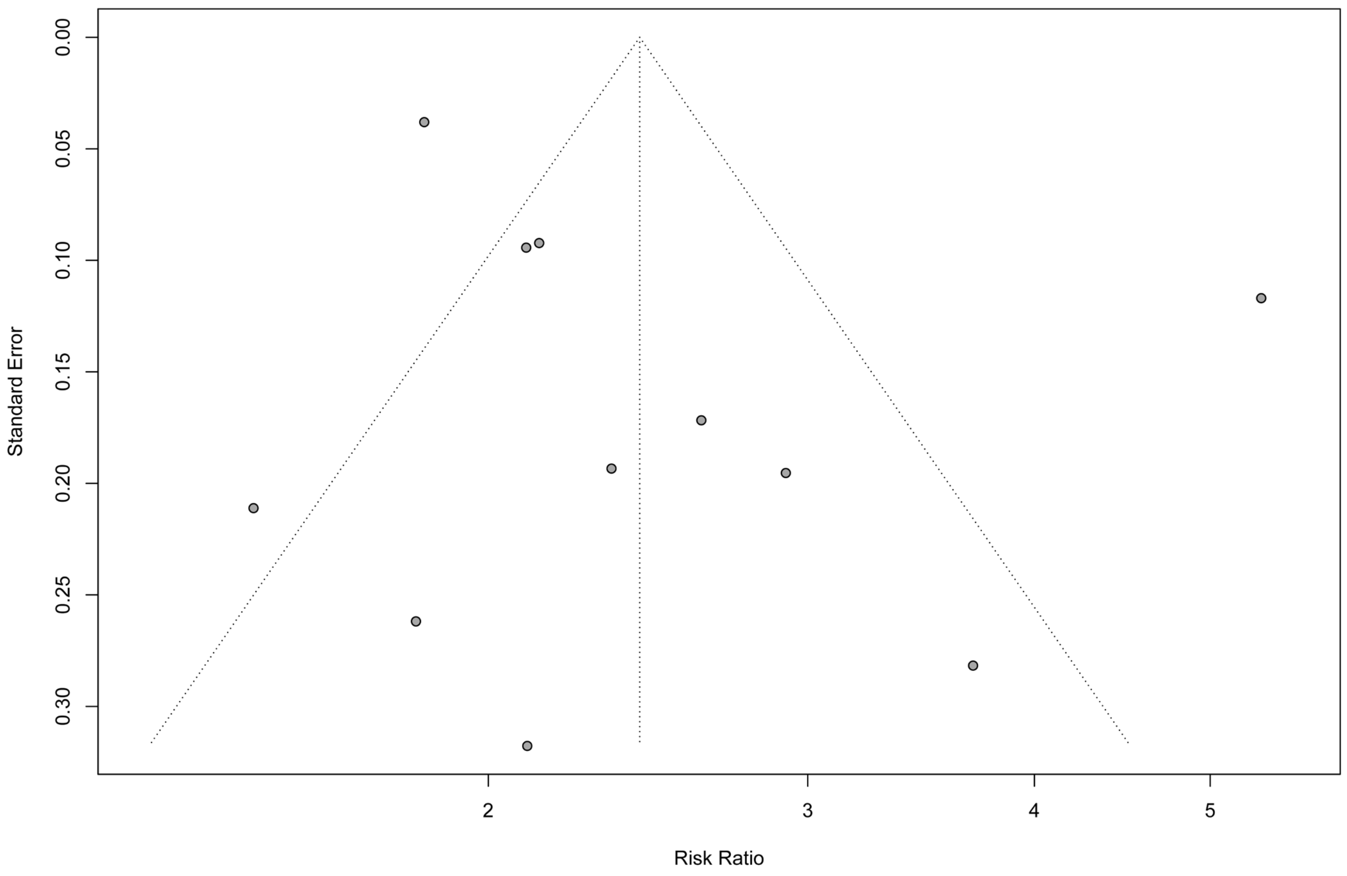

s Fig 7 Peter's test.

```
Linear regression test of funnel plot asymmetry

Test result: t = 1.45, df = 9, p-value = 0.1824

Sample estimates:
   bias se.bias intercept se.intercept
 1.9607  1.3568    0.5881       0.1332

Details:
- multiplicative residual heterogeneity variance (tau^2 = 7.8565)
- predictor: standard error
- weight:    inverse variance
- reference: Egger et al. (1997), BMJ
```

s Fig 8 Subgroup analysis for RAAS blocker users of more or less than 50%.

Study or Subgroup	HUA Events	HUA Total	Common UA Events	Common UA Total	Weight	Risk Ratio MH, Random, 95% CI
RAAS.inhibitors = Less than 50%						
Gang Huang−2018	26	342	29	696	30.6%	1.82 [1.09; 3.05]
Filippo Valbusa et.al−2013	19	73	23	327	27.9%	3.70 [2.13; 6.43]
Total (95% CI)		415		1023	58.5%	2.58 [1.29; 5.16]
Heterogeneity: $Tau^2 = 0.1760$; $Chi^2 = 3.38$, df = 1 (P = 0.07); $I^2 = 70\%$						
RAAS.inhibitors = More than 50%						
A Mantovani et.al−2016	48	233	43	609	41.5%	2.92 [1.99; 4.28]
**Total (95% CI)**		**648**		**1632**	**100.0%**	**2.70 [1.87; 3.90]**
Heterogeneity: $Tau^2 = 0.0468$; $Chi^2 = 3.65$, df = 2 (P = 0.16); $I^2 = 45\%$						
Test for subgroup differences: $Chi^2 = 0.09$, df = 1 (P = 0.76)						

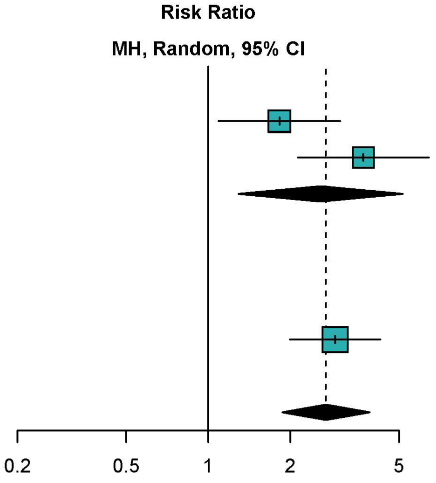

s Fig 9 Meta-regression of eGFR

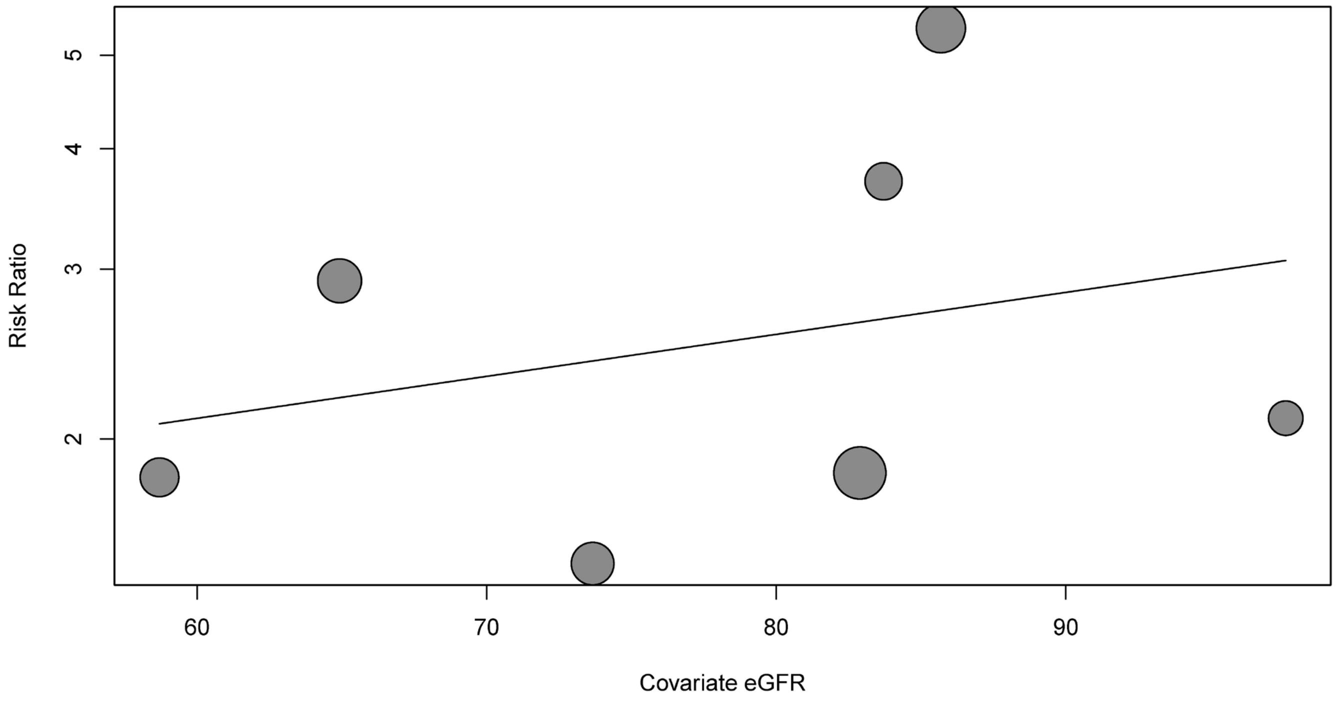

s Table 1. Sensitivity analysis.

Influential analysis (Random effects model)
Risk ratio

	RR	95%-CI
Omitting Gang Huang et.al-2018	2.4792	[1.9557; 3.1428]
Omitting Guo-Zhe Sun et.al-2015	2.4322	[1.9030; 3.1087]
Omitting Yue Chen et.al-2017	2.4472	[1.9287; 3.1051]
Omitting Masanari Kuwabara et.al-2017	2.1354	[1.8904; 2.4122]
Omitting Wei-dong Lin et.al-2019	2.4043	[1.8797; 3.0753]
Omitting S-Y Chuang et.al-2014	2.5350	[2.0213; 3.1794]
Omitting A Mantovani et.al-2016	2.3806	[1.8678; 3.0342]
Omitting Tze-Fan Chao et.al-2013	2.5127	[1.9749; 3.1970]
Omitting Filippo Valbusa et.al-2013	2.3492	[1.8628; 2.9627]
Omitting Leonardo Tamariz-2011	2.4665	[1.9251; 3.1600]
Omitting Hikari Seki, MD et.al-2021	2.4616	[1.9201; 3.1558]
**Pooled estimate**	2.4239	[1.9383; 3.0311]

	p-value	tau^2	tau
Omitting Gang Huang et.al-2018	< 0.0001	0.1156	0.3400
Omitting Guo-Zhe Sun et.al-2015	< 0.0001	0.1232	0.3511
Omitting Yue Chen et.al-2017	< 0.0001	0.1184	0.3441
Omitting Masanari Kuwabara et.al-2017	< 0.0001	0.0145	0.1202
Omitting Wei-dong Lin et.al-2019	< 0.0001	0.1234	0.3513
Omitting S-Y Chuang et.al-2014	< 0.0001	0.1015	0.3186
Omitting A Mantovani et.al-2016	< 0.0001	0.1199	0.3462
Omitting Tze-Fan Chao et.al-2013	< 0.0001	0.1133	0.3365
Omitting Filippo Valbusa et.al-2013	< 0.0001	0.1101	0.3318
Omitting Leonardo Tamariz-2011	< 0.0001	0.1229	0.3506
Omitting Hikari Seki, MD et.al-2021	< 0.0001	0.1237	0.3517
**Pooled estimate**	< 0.0001	0.1097	0.3312

**Heterogeneity**	**I^2**
Omitting Gang Huang et.al-2018	89.6%
Omitting Guo-Zhe Sun et.al-2015	89.6%
Omitting Yue Chen et.al-2017	89.7%
Omitting Masanari Kuwabara et.al-2017	52.0%
Omitting Wei-dong Lin et.al-2019	89.5%
Omitting S-Y Chuang et.al-2014	89.3%
Omitting A Mantovani et.al-2016	89.3%
Omitting Tze-Fan Chao et.al-2013	84.7%
Omitting Filippo Valbusa et.al-2013	89.2%
Omitting Leonardo Tamariz-2011	89.7%
Omitting Hikari Seki, MD et.al-2021	89.7%
Pooled estimate	88.5%

Details on meta-analytical method:
- Mantel-Haenszel method
- Restricted maximum-likelihood estimator for tau^2

s Appendix 1. Search strategy.

Number	Search strategy
#1	Atheromatous Plaques【Title/Abstruct】
#2	Acid, Uric 【Title/Abstruct】
#3	2,6,8-Trihydroxypurine 【Title/Abstruct】
#4	Trioxopurine 【Title/Abstruct】
#5	Potassium Urate 【Title/Abstruct】
#6	Urate, Potassium 【Title/Abstruct】
#7	Urate 【Title/Abstruct】
#8	Ammonium Acid Urate 【Title/Abstruct】
#9	Acid Urate, Ammonium 【Title/Abstruct】
#10	Urate, Ammonium Acid 【Title/Abstruct】
#11	Sodium Urate Monohydrate 【Title/Abstruct】
#12	Monohydrate, Sodium Urate【Title/Abstruct】
#13	Urate Monohydrate, Sodium【Title/Abstruct】
#14	Monosodium Urate Monohydrate【Title/Abstruct】
#15	Monohydrate, Monosodium Urate【Title/Abstruct】
#16	Urate Monohydrate, Monosodium【Title/Abstruct】
#17	Sodium Acid Urate Monohydrate【Title/Abstruct】
#18	Sodium Urate【Title/Abstruct】
#19	Urate, Sodium【Title/Abstruct】
#20	Monosodium Urate【Title/Abstruct】
#21	Urate, Monosodium【Title/Abstruct】
#22	Sodium Acid Urate【Title/Abstruct】
#23	Acid Urate, Sodium【Title/Abstruct】
#24	Urate, Sodium Acid【Title/Abstruct】
#25	#1 or #2 or #3 or #4 or #5 or #6 or #7 or #8 or #9 or #10 or #11 or #12 or #13 or #14 or #15 or #16 or #17 or #18 or #19 or #20 or #21 or #22 or #23 or #24
#26	Atrial Fibrillations【Title/Abstruct】
#27	Fibrillation, Atrial【Title/Abstruct】
#28	Fibrillations, Atrial【Title/Abstruct】
#29	Auricular Fibrillation【Title/Abstruct】
#30	Auricular Fibrillations【Title/Abstruct】
#31	Fibrillation, Auricular【Title/Abstruct】
#32	Fibrillations, Auricular【Title/Abstruct】
#33	Persistent Atrial Fibrillation【Title/Abstruct】
#34	Atrial Fibrillation, Persistent【Title/Abstruct】
#35	Atrial Fibrillations, Persistent【Title/Abstruct】
#36	Fibrillation, Persistent Atrial【Title/Abstruct】
#37	Fibrillations, Persistent Atrial【Title/Abstruct】
#38	Persistent Atrial Fibrillations【Title/Abstruct】
#39	Familial Atrial Fibrillation【Title/Abstruct】
#40	Atrial Fibrillation, Familial【Title/Abstruct】
#41	Atrial Fibrillations, Familial【Title/Abstruct】
#42	Familial Atrial Fibrillations【Title/Abstruct】
#43	Fibrillation, Familial Atrial【Title/Abstruct】
#44	Fibrillations, Familial Atrial【Title/Abstruct】
#45	Paroxysmal Atrial Fibrillation【Title/Abstruct】
#46	Atrial Fibrillation, Paroxysmal【Title/Abstruct】
#47	Atrial Fibrillations, Paroxysmal【Title/Abstruct】
#48	Fibrillation, Paroxysmal Atrial【Title/Abstruct】
#49	Fibrillations, Paroxysmal Atrial【Title/Abstruct】

#50		Paroxysmal Atrial Fibrillations【Title/Abstruct】
#51		#26 or #27 or #28 or #29 or #30 or #31 or #32 or #33 or #34 or #35 or #36 or #37 or #38 or #39 or #40 or #41 or #42 or #43 or #44 or #45 or #46 or #47 or #48 or #49 or #50
#52		Metabolic disorder 【Title/Abstruct】
#53		endocrine disorder 【Title/Abstruct】
#54		#52 or #53
#55		#25 or #54
#56		#55 or #51

Legends:

s Fig 1 summarizes qualities of literatures included in the present review.
s Fig 2 describes risk of biases in each study included.
s Fig 3 describe the meta-regression details of the current drinker's ratio in R studio work station.
s Fig 4A~4F are meta-regression curves of covariates; A, meta-regression of uric acid; B, meta-regression of smoking; C, meta-regression of male proportion; D, meta-regression of BMI; E, meta-regression of current drinking; F, meta-regression of average age.
s Fig 5 is a forest plot of sensitivity analysis of the main outcome.
s Fig 6 intuitively reflects the publication bias.
s Fig 7 linearly quantifies publication bias.
s Fig 8 is the subgroup analysis for RAAS blocker users of more or less than 50%.
S Fig 9 is the meta-regression curve of estimated glomerular filtration rate eGFR
s Table 1 shows details of sensitivity analysis
s Appendix 1 is a comprehensive description of the retrieval strategy used in article identifying.
