# Supplementary material for: Hyperuricemia Increases the Risk of Atrial Fibrillation: A Systematic Review and Meta-Analysis
Source: Int J Endocrinol. 2022 Aug 21;2022:8172639. doi: 10.1155/2022/8172639 (PMC9420608; doi:10.1155/2022/8172639)
Supplement: Supplementary Materials — 1. Supplements. To make our work more organized, some pictures or tables were put in a supplementary material named Supplements. All the pictures and tables in the Supplements were cited and illustrated in the article. 2. Graphical Abstract Image. A graphical abstract, concise and comprehensive of the main contribution of our work. 3. Graphical Abstract Text. A short explanation of our graphical abstract. [file 8172639.f1.zip › Supplements, s Appendix 1.docx]

s Appendix 1. Search strategy.

| **Search strategy** | |
| --- | --- |
| Number |  |
| #1 | Atheromatous Plaques【Title/Abstruct】 |
| #2 | Acid, Uric 【Title/Abstruct】 |
| #3 | 2,6,8-Trihydroxypurine 【Title/Abstruct】 |
| #4 | Trioxopurine 【Title/Abstruct】 |
| #5 | Potassium Urate 【Title/Abstruct】 |
| #6 | Urate, Potassium 【Title/Abstruct】 |
| #7 | Urate 【Title/Abstruct】 |
| #8 | Ammonium Acid Urate 【Title/Abstruct】 |
| #9 | Acid Urate, Ammonium 【Title/Abstruct】 |
| #10 | Urate, Ammonium Acid 【Title/Abstruct】 |
| #11 | Sodium Urate Monohydrate 【Title/Abstruct】 |
| #12 | Monohydrate, Sodium Urate【Title/Abstruct】 |
| #13 | Urate Monohydrate, Sodium【Title/Abstruct】 |
| #14 | Monosodium Urate Monohydrate【Title/Abstruct】 |
| #15 | Monohydrate, Monosodium Urate【Title/Abstruct】 |
| #16 | Urate Monohydrate, Monosodium【Title/Abstruct】 |
| #17 | Sodium Acid Urate Monohydrate【Title/Abstruct】 |
| #18 | Sodium Urate【Title/Abstruct】 |
| #19 | Urate, Sodium【Title/Abstruct】 |
| #20 | Monosodium Urate【Title/Abstruct】 |
| #21 | Urate, Monosodium【Title/Abstruct】 |
| #22 | Sodium Acid Urate【Title/Abstruct】 |
| #23 | Acid Urate, Sodium【Title/Abstruct】 |
| #24 | Urate, Sodium Acid【Title/Abstruct】 |
| #25 | #1 or #2 or #3 or #4 or #5 or #6 or #7 or #8 or #9 or #10 or #11 or #12 or #13 or #14 or #15 or #16 or #17 or #18 or #19 or #20 or #21 or #22 or #23 or #24 |
| #26 | Atrial Fibrillations【Title/Abstruct】 |
| #27 | Fibrillation, Atrial【Title/Abstruct】 |
| #28 | Fibrillations, Atrial【Title/Abstruct】 |
| #29 | Auricular Fibrillation【Title/Abstruct】 |
| #30 | Auricular Fibrillations【Title/Abstruct】 |
| #31 | Fibrillation, Auricular【Title/Abstruct】 |
| #32 | Fibrillations, Auricular【Title/Abstruct】 |
| #33 | Persistent Atrial Fibrillation【Title/Abstruct】 |
| #34 | Atrial Fibrillation, Persistent【Title/Abstruct】 |
| #35 | Atrial Fibrillations, Persistent【Title/Abstruct】 |
| #36 | Fibrillation, Persistent Atrial【Title/Abstruct】 |
| #37 | Fibrillations, Persistent Atrial【Title/Abstruct】 |
| #38 | Persistent Atrial Fibrillations【Title/Abstruct】 |
| #39 | Familial Atrial Fibrillation【Title/Abstruct】 |
| #40 | Atrial Fibrillation, Familial【Title/Abstruct】 |
| #41 | Atrial Fibrillations, Familial【Title/Abstruct】 |
| #42 | Familial Atrial Fibrillations【Title/Abstruct】 |
| #43 | Fibrillation, Familial Atrial【Title/Abstruct】 |
| #44 | Fibrillations, Familial Atrial【Title/Abstruct】 |
| #45 | Paroxysmal Atrial Fibrillation【Title/Abstruct】 |
| #46 | Atrial Fibrillation, Paroxysmal【Title/Abstruct】 |
| #47 | Atrial Fibrillations, Paroxysmal【Title/Abstruct】 |
| #48 | Fibrillation, Paroxysmal Atrial【Title/Abstruct】 |
| #49 | Fibrillations, Paroxysmal Atrial【Title/Abstruct】 |
| #50 | Paroxysmal Atrial Fibrillations【Title/Abstruct】 |
| #51 | #26 or #27 or #28 or #29 or #30 or #31 or #32 or #33 or #34 or #35 or #36 or #37 or #38 or #39 or #40 or #41 or #42 or #43 or #44 or #45 or #46 or #47 or #48 or #49 or #50 |
| #52 | Metabolic disorder 【Title/Abstruct】 |
| #53 | endocrine disorder 【Title/Abstruct】 |
| #54 | #52 or #53 |
| #55 | #25 or #54 |
| #56 | #55 or #51 |
