# Supplementary material for: Hyperuricemia Increases the Risk of Atrial Fibrillation: A Systematic Review and Meta-Analysis
Source: Int J Endocrinol. 2022 Aug 21;2022:8172639. doi: 10.1155/2022/8172639 (PMC9420608; doi:10.1155/2022/8172639)
Supplement: Supplementary Materials — 1. Supplements. To make our work more organized, some pictures or tables were put in a supplementary material named Supplements. All the pictures and tables in the Supplements were cited and illustrated in the article. 2. Graphical Abstract Image. A graphical abstract, concise and comprehensive of the main contribution of our work. 3. Graphical Abstract Text. A short explanation of our graphical abstract. [file 8172639.f1.zip › Supplements, s Figure 3.pdf]

```
Mixed-Effects Model (k = 5; tau^2 estimator: REML)

tau^2 (estimated amount of residual heterogeneity):     0 (SE = 0.0232)
tau (square root of estimated tau^2 value):             0
I^2 (residual heterogeneity / unaccounted variability): 0.00%
H^2 (unaccounted variability / sampling variability):   1.00
R^2 (amount of heterogeneity accounted for):            100.00%

Test for Residual Heterogeneity:
QE(df = 3) = 1.6346, p-val = 0.6516

Test of Moderators (coefficient 2):
QM(df = 1) = 41.0069, p-val < .0001

Model Results:

          estimate      se    zval    pval   ci.lb   ci.ub
intrcpt     0.4180  0.1167  3.5831  0.0003  0.1893  0.6466  ***
Drinking    2.0054  0.3132  6.4037  <.0001  1.3916  2.6192  ***

---
Signif. codes:  0 '***' 0.001 '**' 0.01 '*' 0.05 '.' 0.1 ' ' 1
```
