# Supplementary material for: Hyperuricemia Increases the Risk of Atrial Fibrillation: A Systematic Review and Meta-Analysis
Source: Int J Endocrinol. 2022 Aug 21;2022:8172639. doi: 10.1155/2022/8172639 (PMC9420608; doi:10.1155/2022/8172639)
Supplement: Supplementary Materials — 1. Supplements. To make our work more organized, some pictures or tables were put in a supplementary material named Supplements. All the pictures and tables in the Supplements were cited and illustrated in the article. 2. Graphical Abstract Image. A graphical abstract, concise and comprehensive of the main contribution of our work. 3. Graphical Abstract Text. A short explanation of our graphical abstract. [file 8172639.f1.zip › Supplements, s Figure 7.pdf]

```
Linear regression test of funnel plot asymmetry

Test result: t = 1.45, df = 9, p-value = 0.1824

Sample estimates:
   bias se.bias intercept se.intercept
 1.9607  1.3568    0.5881       0.1332

Details:
- multiplicative residual heterogeneity variance (tau^2 = 7.8565)
- predictor: standard error
- weight:    inverse variance
- reference: Egger et al. (1997), BMJ
```
