# Supplementary material for: Hyperuricemia Increases the Risk of Atrial Fibrillation: A Systematic Review and Meta-Analysis
Source: Int J Endocrinol. 2022 Aug 21;2022:8172639. doi: 10.1155/2022/8172639 (PMC9420608; doi:10.1155/2022/8172639)

Study or Subgroup	HUA Events	HUA Total	Common UA Events	Common UA Total	Weight	Risk Ratio MH, Random, 95% CI
RAAS.inhibitors = Less than 50%						
Gang Huang−2018	26	342	29	696	30.6%	1.82 [1.09; 3.05]
Filippo Valbusa et.al−2013	19	73	23	327	27.9%	3.70 [2.13; 6.43]
Total (95% CI)		415		1023	58.5%	2.58 [1.29; 5.16]
Heterogeneity: $Tau^2 = 0.1760$; $Chi^2 = 3.38$, df = 1 (P = 0.07); $I^2 = 70\%$						
RAAS.inhibitors = More than 50%						
A Mantovani et.al−2016	48	233	43	609	41.5%	2.92 [1.99; 4.28]
**Total (95% CI)**		**648**		**1632**	**100.0%**	**2.70 [1.87; 3.90]**

Heterogeneity: $Tau^2 = 0.0468$; $Chi^2 = 3.65$, df = 2 (P = 0.16); $I^2 = 45\%$

Test for subgroup differences: $Chi^2 = 0.09$, df = 1 (P = 0.76)

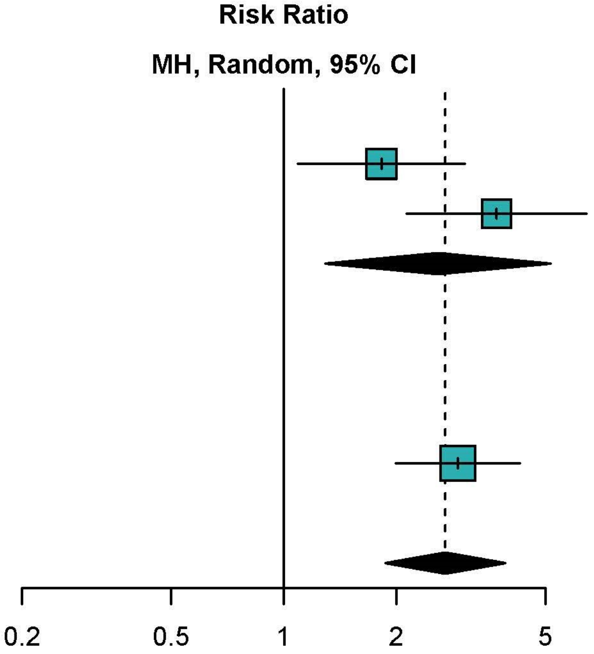

Supplement: Supplementary Materials — 1. Supplements. To make our work more organized, some pictures or tables were put in a supplementary material named Supplements. All the pictures and tables in the Supplements were cited and illustrated in the article. 2. Graphical Abstract Image. A graphical abstract, concise and comprehensive of the main contribution of our work. 3. Graphical Abstract Text. A short explanation of our graphical abstract. [file 8172639.f1.zip › Supplements, s Figure 8.pdf]
