# Supplementary material for: Hyperuricemia Increases the Risk of Atrial Fibrillation: A Systematic Review and Meta-Analysis
Source: Int J Endocrinol. 2022 Aug 21;2022:8172639. doi: 10.1155/2022/8172639 (PMC9420608; doi:10.1155/2022/8172639)
Supplement: Supplementary Materials — 1. Supplements. To make our work more organized, some pictures or tables were put in a supplementary material named Supplements. All the pictures and tables in the Supplements were cited and illustrated in the article. 2. Graphical Abstract Image. A graphical abstract, concise and comprehensive of the main contribution of our work. 3. Graphical Abstract Text. A short explanation of our graphical abstract. [file 8172639.f1.zip › Supplements, s Table 1.docx]

s Table 1. Sensitivity analysis.

| **Influential analysis (Random effects model)** | | | |
| --- | --- | --- | --- |
| **Risk ratio** |  | | |
|  | **RR** | **95%-CI** | |
| Omitting Gang Huang et.al-2018 | 2.4792 | [1.9557; 3.1428] | |
| Omitting Guo-Zhe Sun et.al-2015 | 2.4322 | [1.9030; 3.1087] | |
| Omitting Yue Chen et.al-2017 | 2.4472 | [1.9287; 3.1051] | |
| Omitting Masanari Kuwabara et.al-2017 | 2.1354 | [1.8904; 2.4122] | |
| Omitting Wei-dong Lin et.al-2019 | 2.4043 | [1.8797; 3.0753] | |
| Omitting S-Y Chuang et.al-2014 | 2.5350 | [2.0213; 3.1794] | |
| Omitting A Mantovani et.al-2016 | 2.3806 | [1.8678; 3.0342] | |
| Omitting Tze-Fan Chao et.al-2013 | 2.5127 | [1.9749; 3.1970] | |
| Omitting Filippo Valbusa et.al-2013 | 2.3492 | [1.8628; 2.9627] | |
| Omitting Leonardo Tamariz-2011 | 2.4665 | [1.9251; 3.1600] | |
| Omitting Hikari Seki, MD et.al-2021 | 2.4616 | [1.9201; 3.1558] | |
|  | | | |
| **Pooled estimate** | 2.4239 | [1.9383; 3.0311] | |
|  | p-value | tau^2 tau | |
| Omitting Gang Huang et.al-2018 | < 0.0001 | 0.1156 | 0.3400 |
| Omitting Guo-Zhe Sun et.al-2015 | < 0.0001 | 0.1232 | 0.3511 |
| Omitting Yue Chen et.al-2017 | < 0.0001 | 0.1184 | 0.3441 |
| Omitting Masanari Kuwabara et.al-2017 | < 0.0001 | 0.0145 | 0.1202 |
| Omitting Wei-dong Lin et.al-2019 | < 0.0001 | 0.1234 | 0.3513 |
| Omitting S-Y Chuang et.al-2014 | < 0.0001 | 0.1015 | 0.3186 |
| Omitting A Mantovani et.al-2016 | < 0.0001 | 0.1199 | 0.3462 |
| Omitting Tze-Fan Chao et.al-2013 | < 0.0001 | 0.1133 | 0.3365 |
| Omitting Filippo Valbusa et.al-2013 | < 0.0001 | 0.1101 | 0.3318 |
| Omitting Leonardo Tamariz-2011 | < 0.0001 | 0.1229 | 0.3506 |
| Omitting Hikari Seki, MD et.al-2021 | < 0.0001 | 0.1237 | 0.3517 |
|  | | | |
| **Pooled estimate** | < 0.0001 | 0.1097 | 0.3312 |
| **Heterogeneity** | **I^2** | | |
| Omitting Gang Huang et.al-2018 | 89.6% | | |
| Omitting Guo-Zhe Sun et.al-2015 | 89.6% | | |
| Omitting Yue Chen et.al-2017 | 89.7% | | |
| Omitting Masanari Kuwabara et.al-2017 | 52.0% | | |
| Omitting Wei-dong Lin et.al-2019 | 89.5% | | |
| Omitting S-Y Chuang et.al-2014 | 89.3% | | |
| Omitting A Mantovani et.al-2016 | 89.3% | | |
| Omitting Tze-Fan Chao et.al-2013 | 84.7% | | |
| Omitting Filippo Valbusa et.al-2013 | 89.2% | | |
| Omitting Leonardo Tamariz-2011 | 89.7% | | |
| Omitting Hikari Seki, MD et.al-2021 | 89.7% | | |
|  | | | |
| Pooled estimate 88.5% | | | |
|  | | | |
| Details on meta-analytical method: | | | |
| - Mantel-Haenszel method | | | |
| - Restricted maximum-likelihood estimator for tau^2 | | | |
