# Supplementary figures and images for: Hyperuricemia Increases the Risk of Atrial Fibrillation: A Systematic Review and Meta-Analysis
Source: Int J Endocrinol. 2022 Aug 21;2022:8172639. doi: 10.1155/2022/8172639 (PMC9420608; doi:10.1155/2022/8172639)

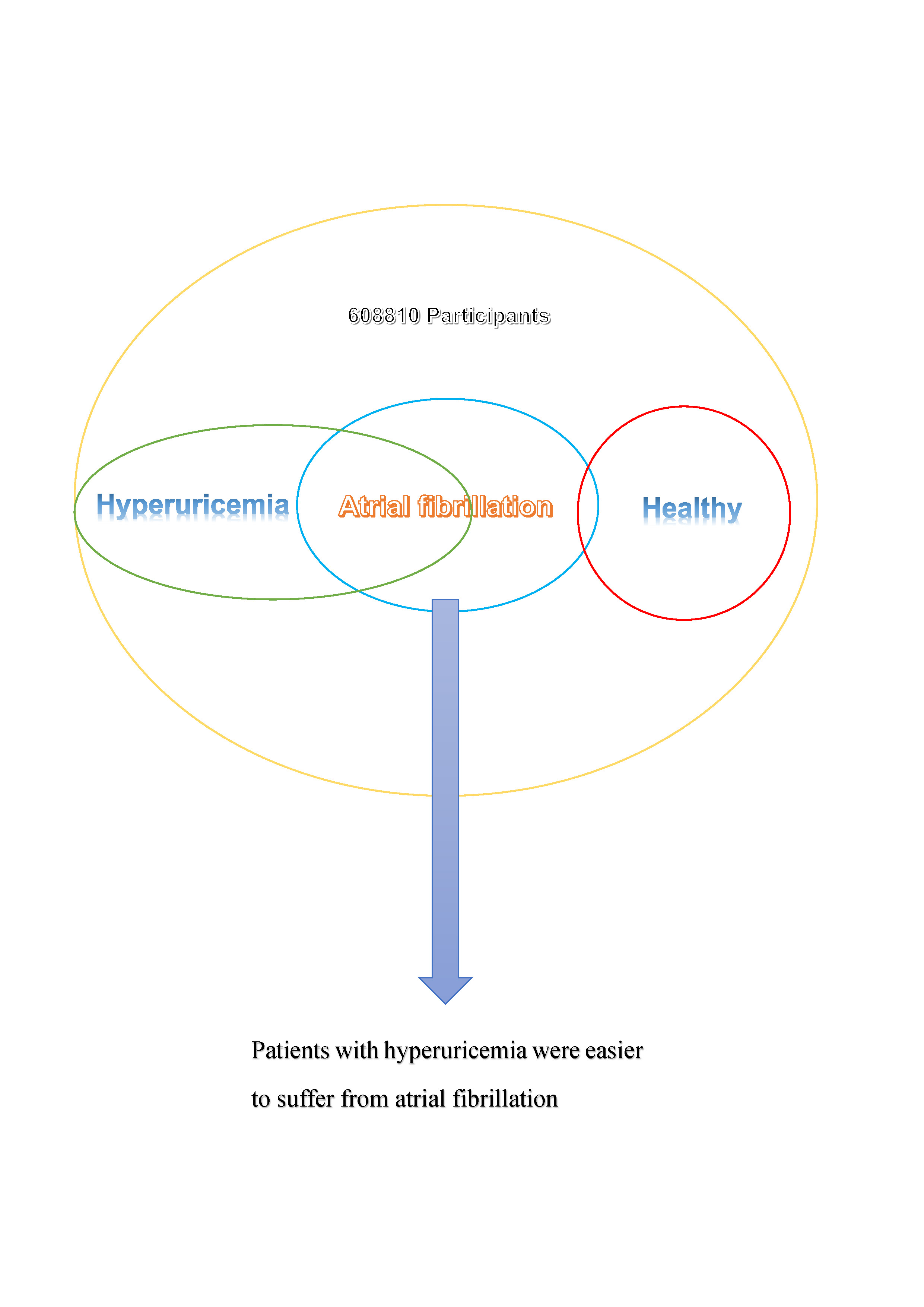

Supplement: Supplementary Materials — 1. Supplements. To make our work more organized, some pictures or tables were put in a supplementary material named Supplements. All the pictures and tables in the Supplements were cited and illustrated in the article. 2. Graphical Abstract Image. A graphical abstract, concise and comprehensive of the main contribution of our work. 3. Graphical Abstract Text. A short explanation of our graphical abstract. [file 8172639.f1.zip › Graphical_Abstract_Image.jpg]

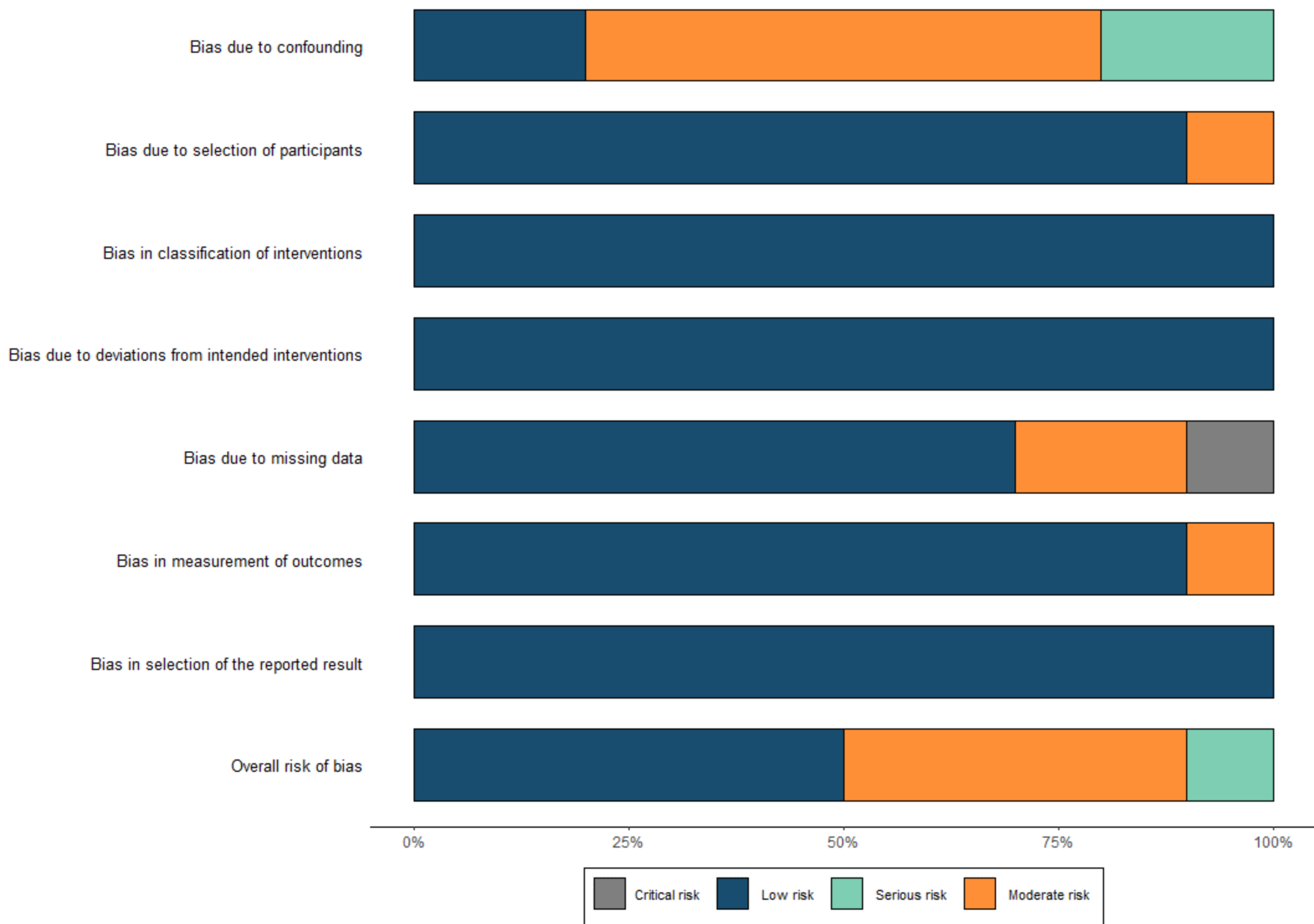

Supplement: Supplementary Materials — 1. Supplements. To make our work more organized, some pictures or tables were put in a supplementary material named Supplements. All the pictures and tables in the Supplements were cited and illustrated in the article. 2. Graphical Abstract Image. A graphical abstract, concise and comprehensive of the main contribution of our work. 3. Graphical Abstract Text. A short explanation of our graphical abstract. [file 8172639.f1.zip › Supplements, s Figure 1.pdf]

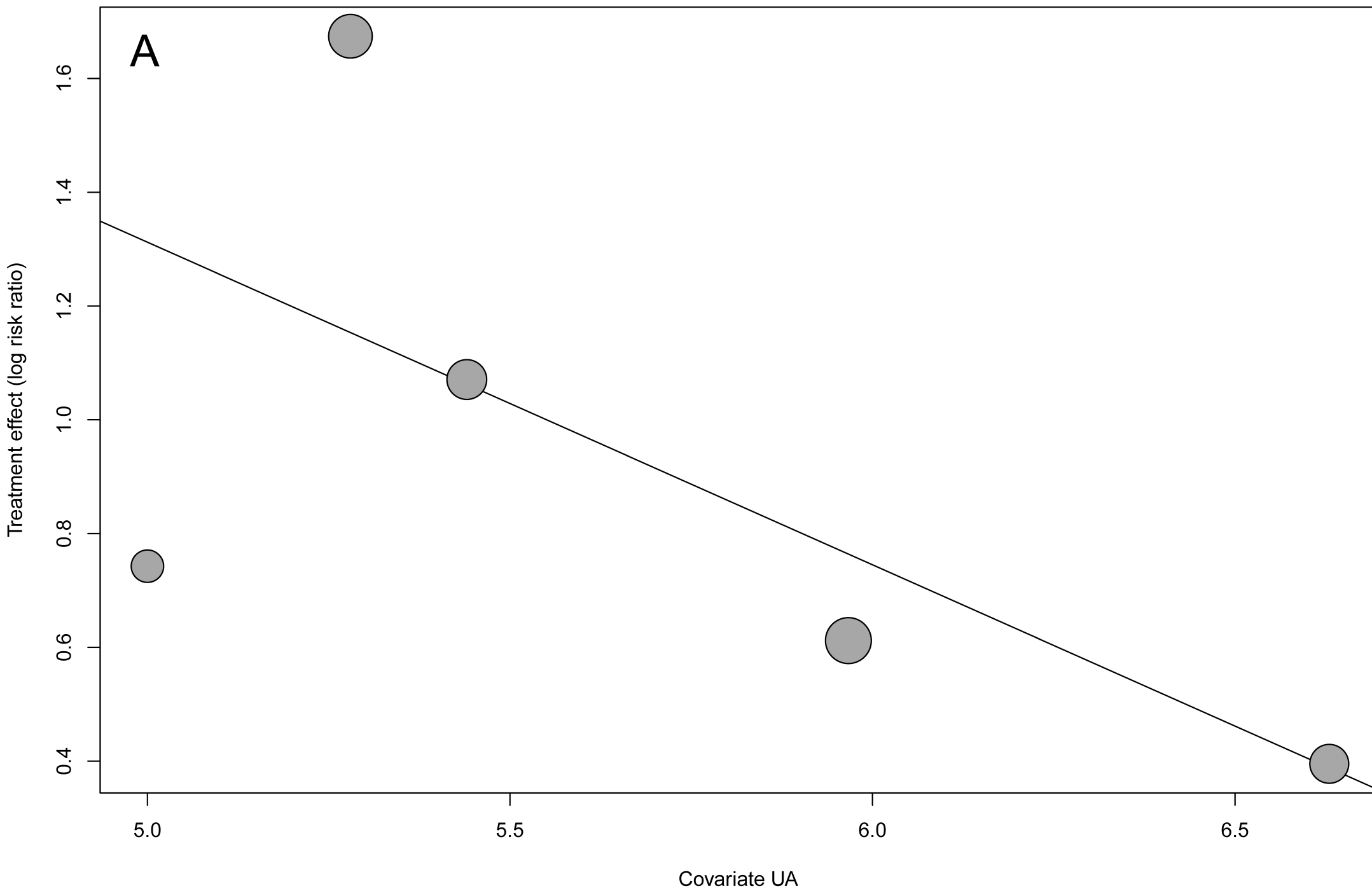

Supplement: Supplementary Materials — 1. Supplements. To make our work more organized, some pictures or tables were put in a supplementary material named Supplements. All the pictures and tables in the Supplements were cited and illustrated in the article. 2. Graphical Abstract Image. A graphical abstract, concise and comprehensive of the main contribution of our work. 3. Graphical Abstract Text. A short explanation of our graphical abstract. [file 8172639.f1.zip › Supplements, s Figure 4A.pdf]

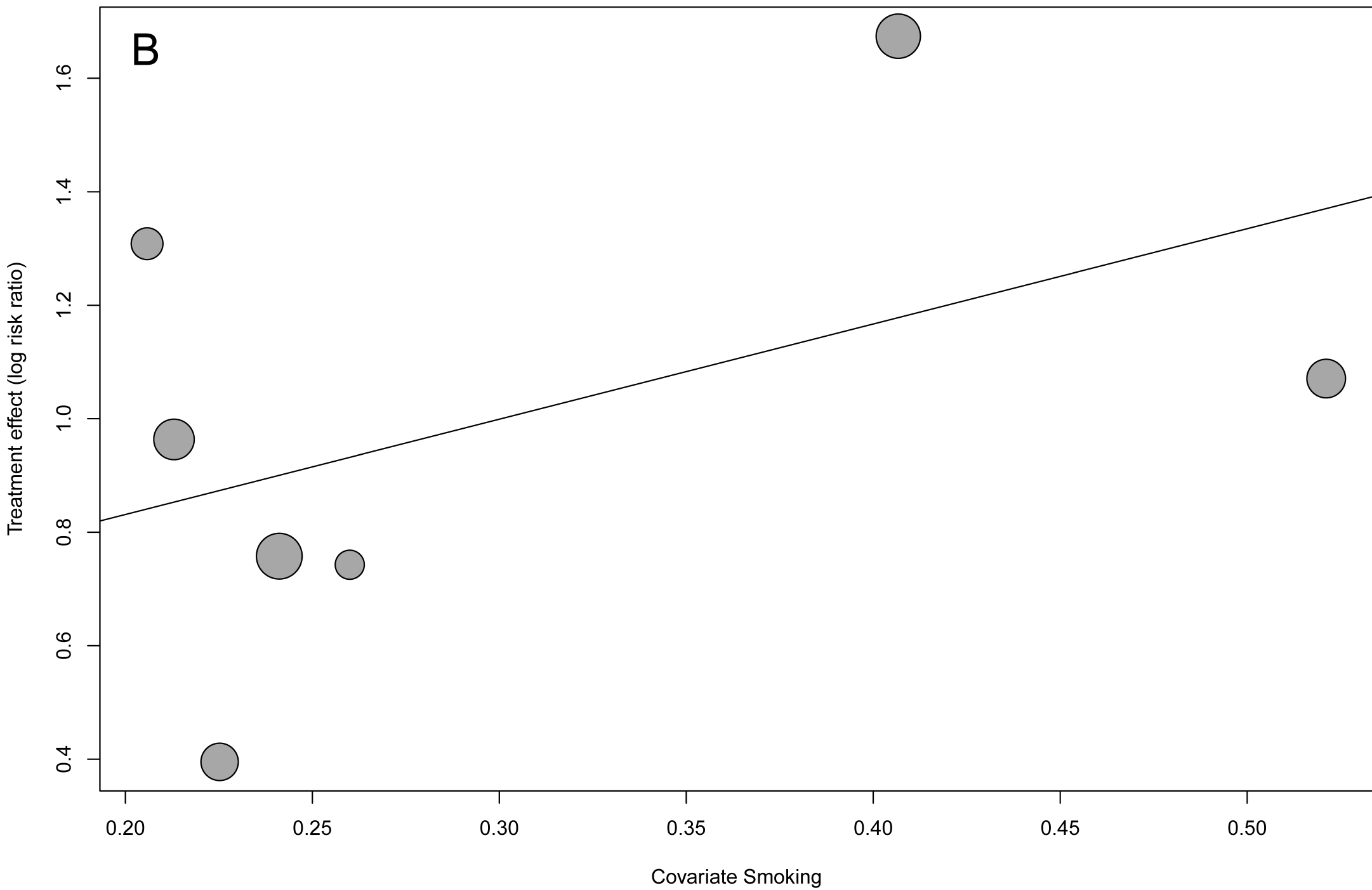

Supplement: Supplementary Materials — 1. Supplements. To make our work more organized, some pictures or tables were put in a supplementary material named Supplements. All the pictures and tables in the Supplements were cited and illustrated in the article. 2. Graphical Abstract Image. A graphical abstract, concise and comprehensive of the main contribution of our work. 3. Graphical Abstract Text. A short explanation of our graphical abstract. [file 8172639.f1.zip › Supplements, s Figure 4B.pdf]

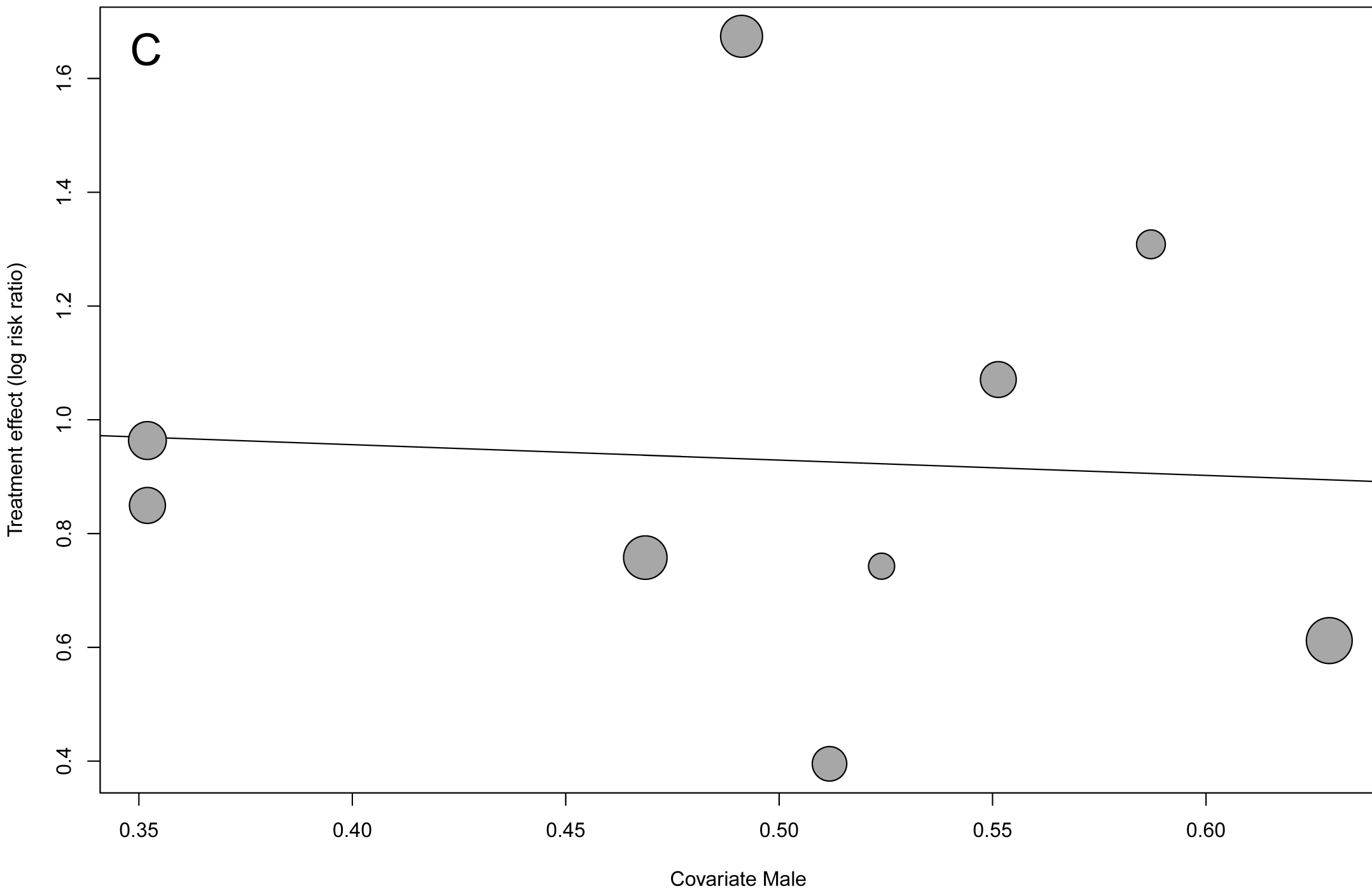

Supplement: Supplementary Materials — 1. Supplements. To make our work more organized, some pictures or tables were put in a supplementary material named Supplements. All the pictures and tables in the Supplements were cited and illustrated in the article. 2. Graphical Abstract Image. A graphical abstract, concise and comprehensive of the main contribution of our work. 3. Graphical Abstract Text. A short explanation of our graphical abstract. [file 8172639.f1.zip › Supplements, s Figure 4C.pdf]

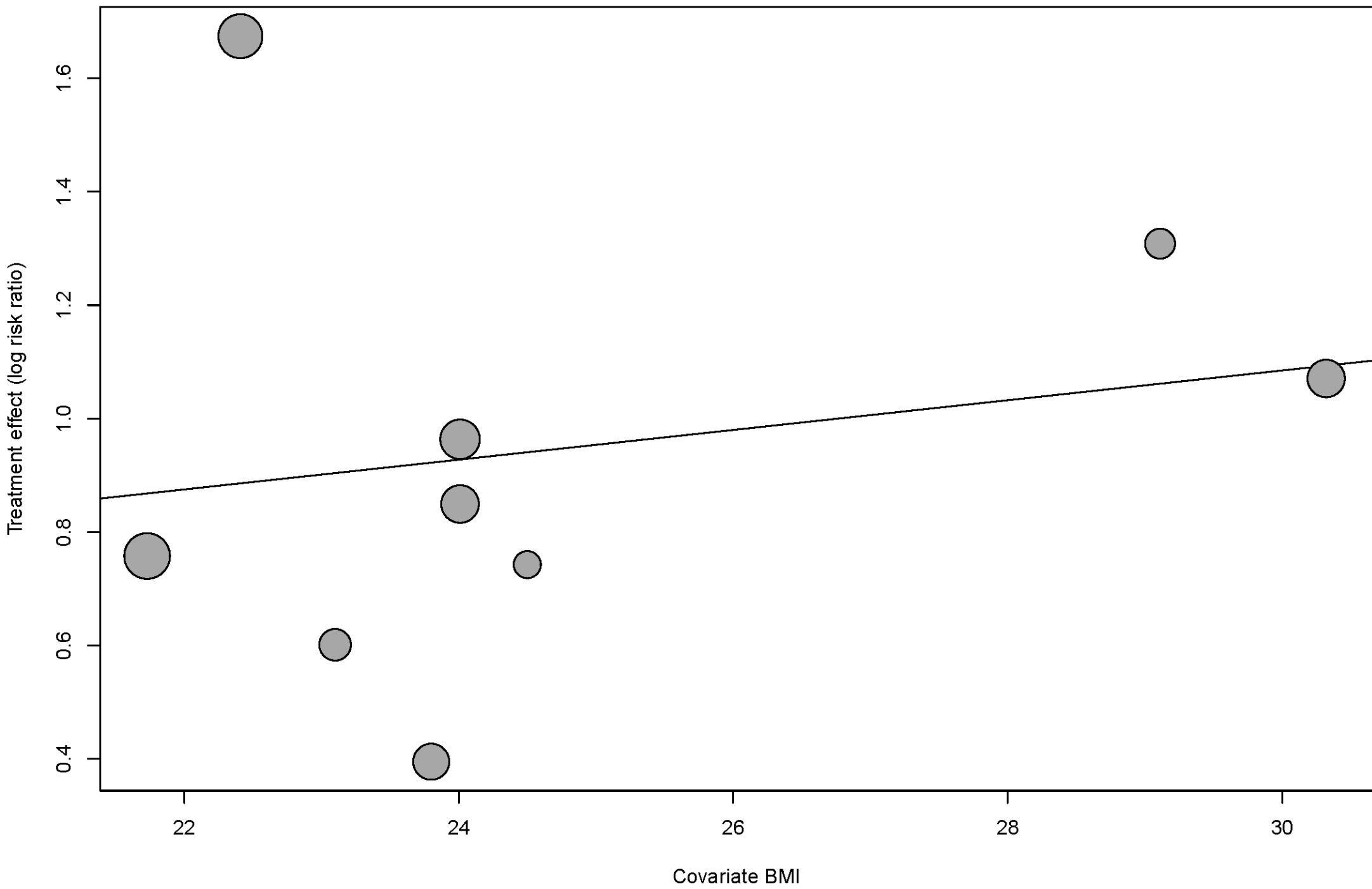

Supplement: Supplementary Materials — 1. Supplements. To make our work more organized, some pictures or tables were put in a supplementary material named Supplements. All the pictures and tables in the Supplements were cited and illustrated in the article. 2. Graphical Abstract Image. A graphical abstract, concise and comprehensive of the main contribution of our work. 3. Graphical Abstract Text. A short explanation of our graphical abstract. [file 8172639.f1.zip › Supplements, s Figure 4D.pdf]

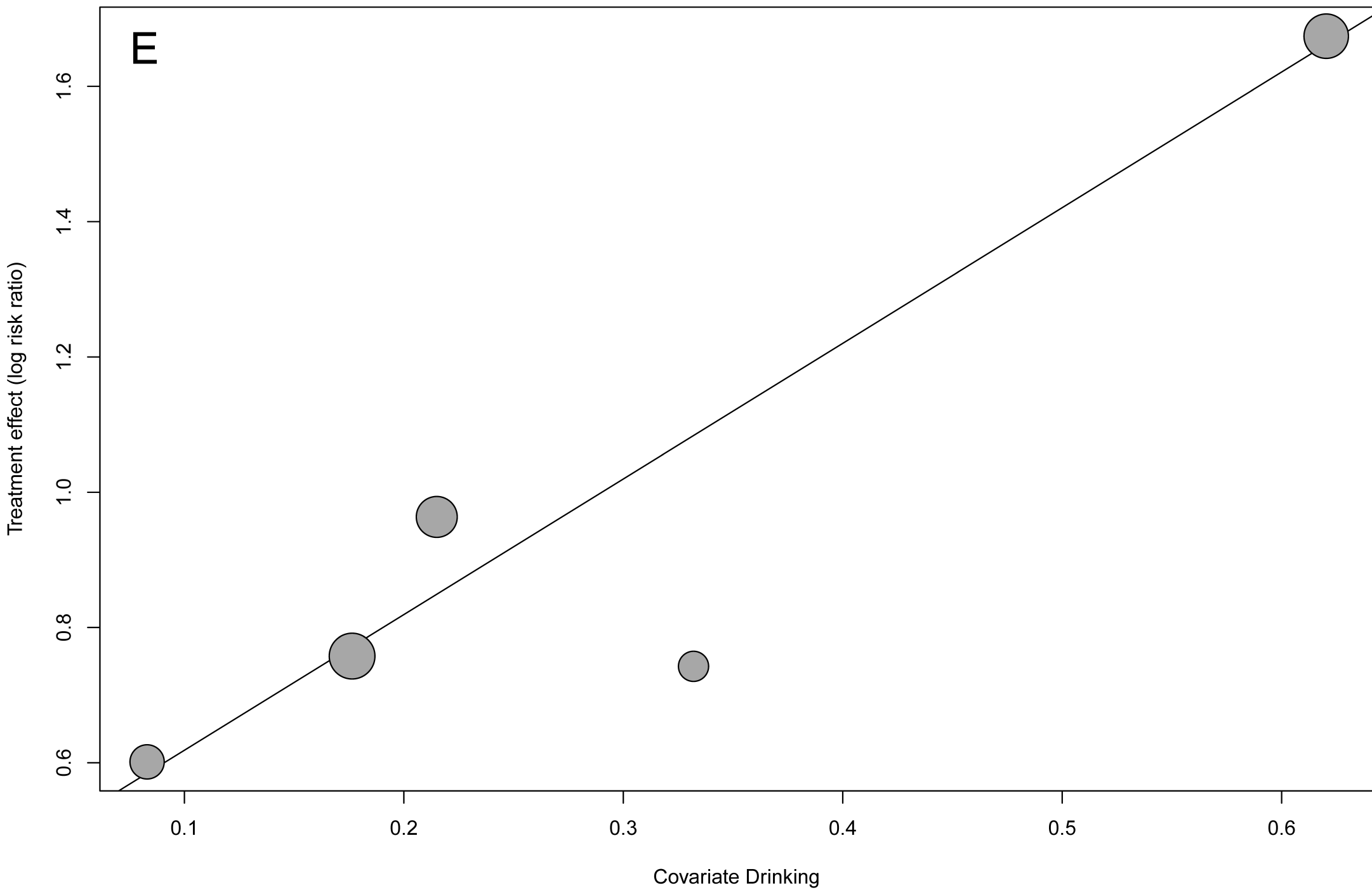

Supplement: Supplementary Materials — 1. Supplements. To make our work more organized, some pictures or tables were put in a supplementary material named Supplements. All the pictures and tables in the Supplements were cited and illustrated in the article. 2. Graphical Abstract Image. A graphical abstract, concise and comprehensive of the main contribution of our work. 3. Graphical Abstract Text. A short explanation of our graphical abstract. [file 8172639.f1.zip › Supplements, s Figure 4E.pdf]

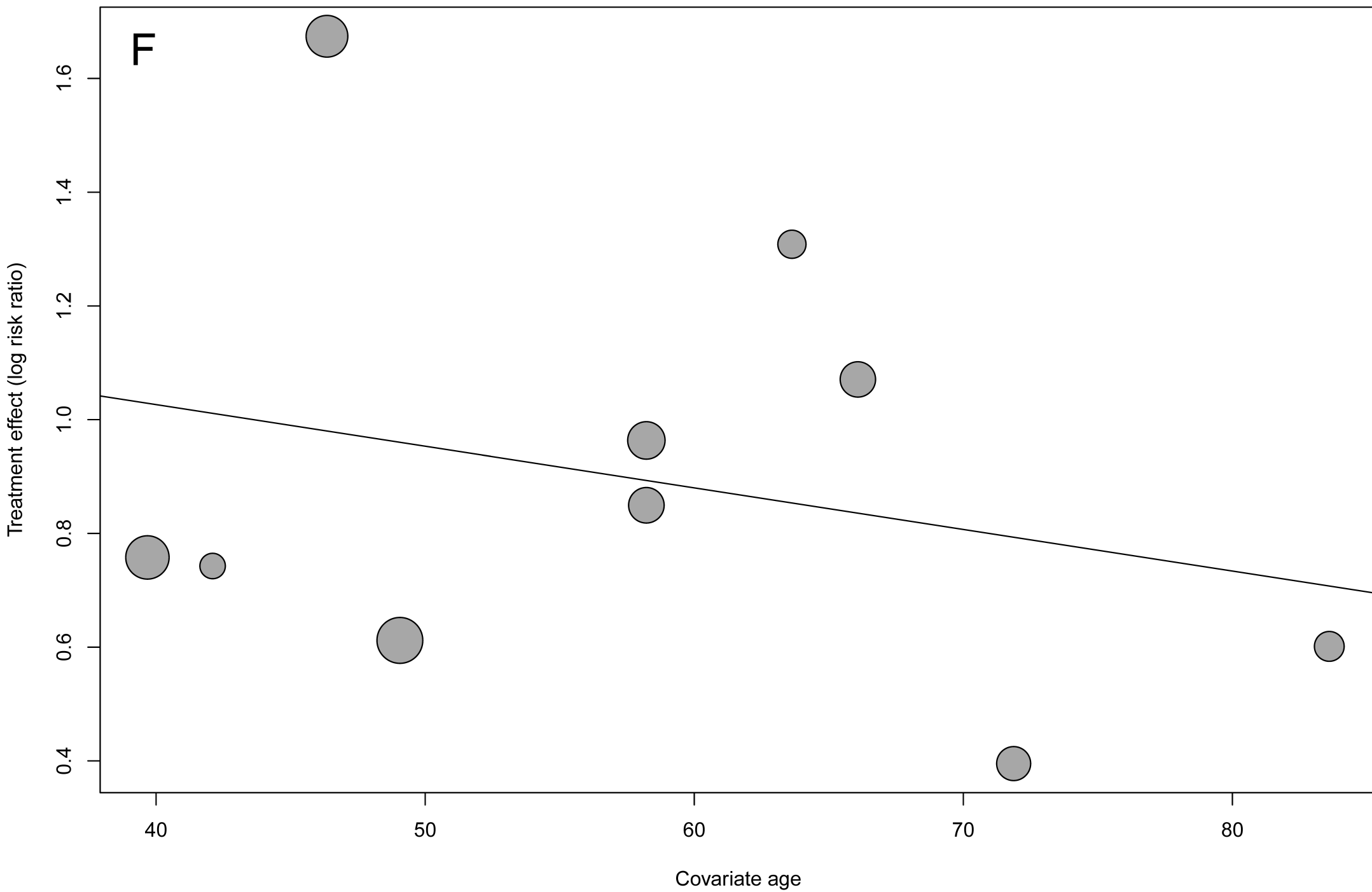

Supplement: Supplementary Materials — 1. Supplements. To make our work more organized, some pictures or tables were put in a supplementary material named Supplements. All the pictures and tables in the Supplements were cited and illustrated in the article. 2. Graphical Abstract Image. A graphical abstract, concise and comprehensive of the main contribution of our work. 3. Graphical Abstract Text. A short explanation of our graphical abstract. [file 8172639.f1.zip › Supplements, s Figure 4F.pdf]

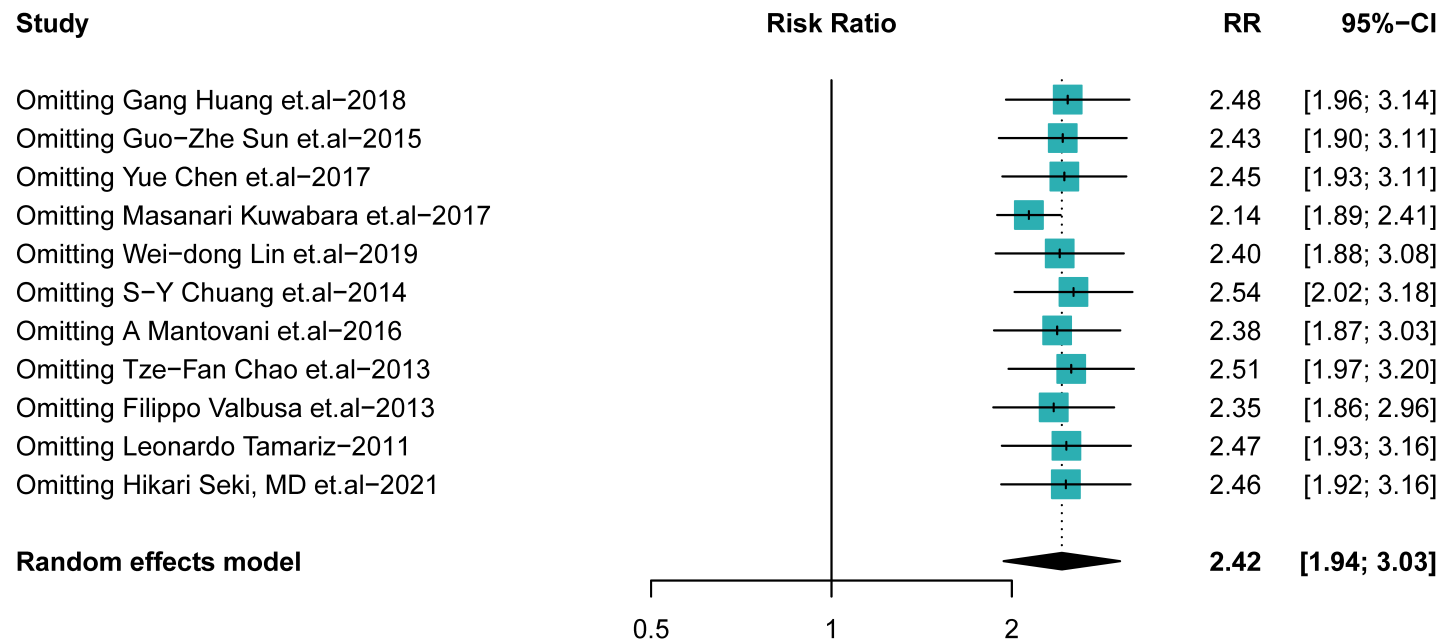

Supplement: Supplementary Materials — 1. Supplements. To make our work more organized, some pictures or tables were put in a supplementary material named Supplements. All the pictures and tables in the Supplements were cited and illustrated in the article. 2. Graphical Abstract Image. A graphical abstract, concise and comprehensive of the main contribution of our work. 3. Graphical Abstract Text. A short explanation of our graphical abstract. [file 8172639.f1.zip › Supplements, s Figure 5.pdf]

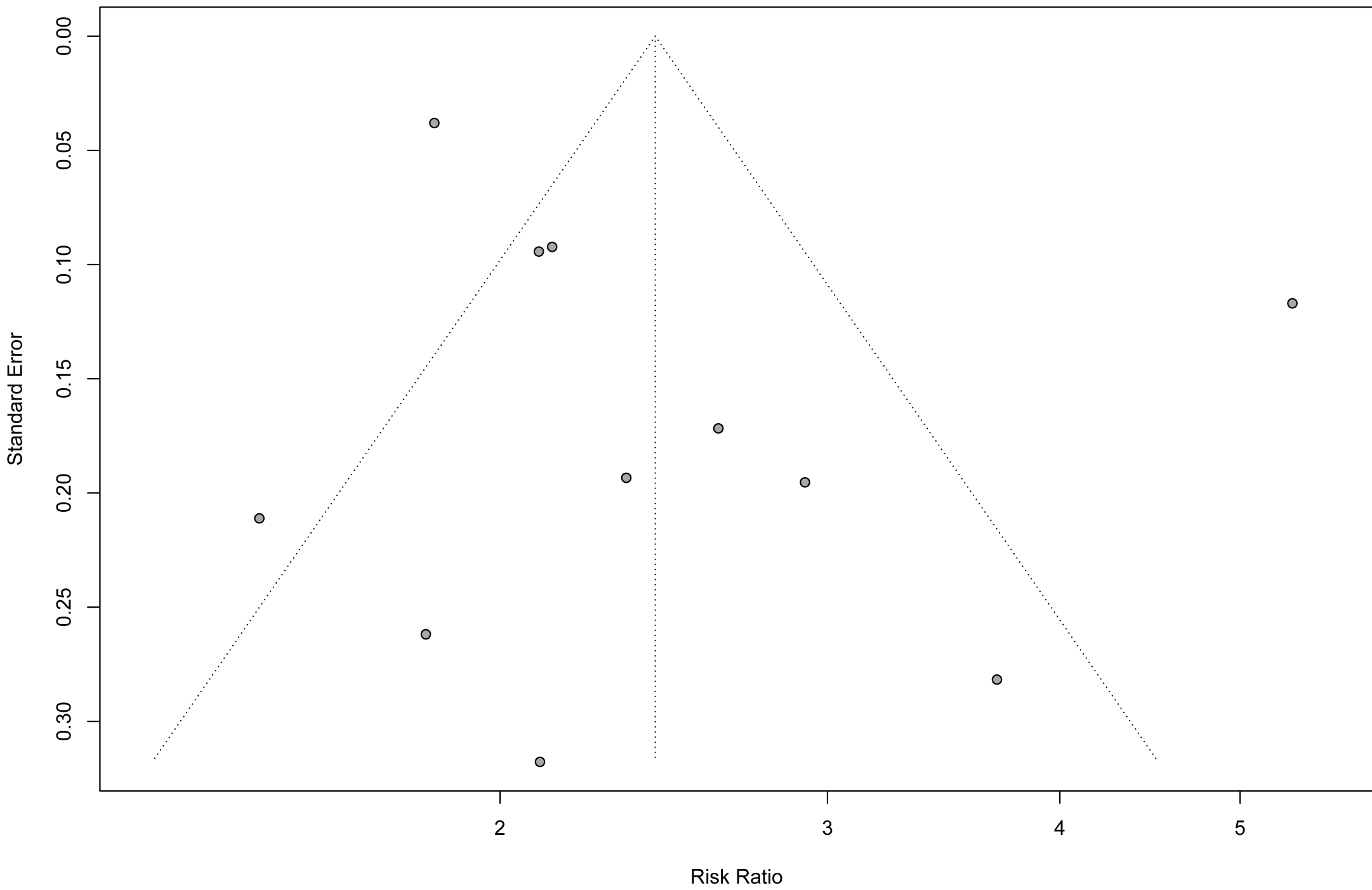

Supplement: Supplementary Materials — 1. Supplements. To make our work more organized, some pictures or tables were put in a supplementary material named Supplements. All the pictures and tables in the Supplements were cited and illustrated in the article. 2. Graphical Abstract Image. A graphical abstract, concise and comprehensive of the main contribution of our work. 3. Graphical Abstract Text. A short explanation of our graphical abstract. [file 8172639.f1.zip › Supplements, s Figure 6.pdf]

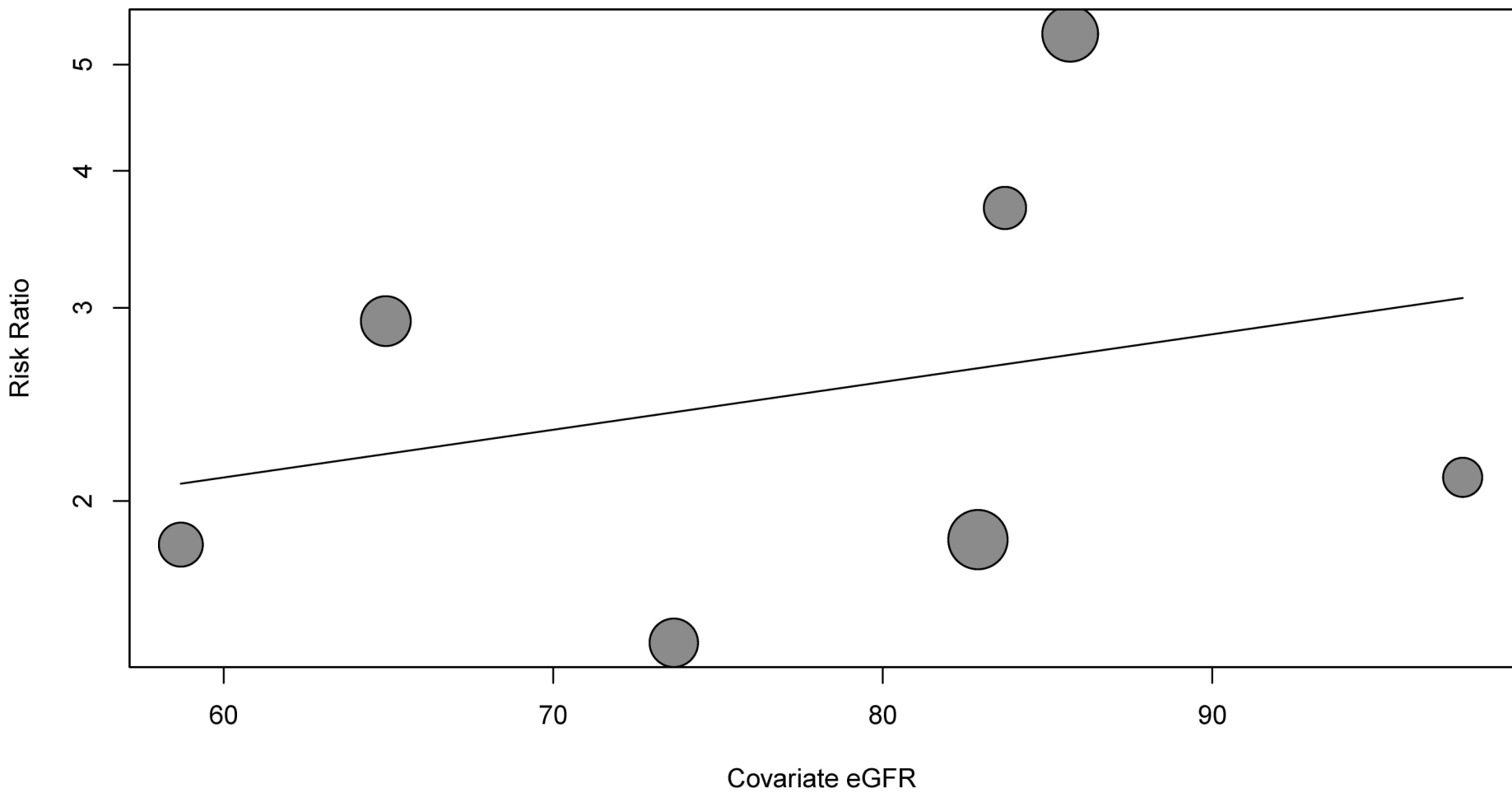

Supplement: Supplementary Materials — 1. Supplements. To make our work more organized, some pictures or tables were put in a supplementary material named Supplements. All the pictures and tables in the Supplements were cited and illustrated in the article. 2. Graphical Abstract Image. A graphical abstract, concise and comprehensive of the main contribution of our work. 3. Graphical Abstract Text. A short explanation of our graphical abstract. [file 8172639.f1.zip › Supplements, s Figure 9.pdf]
